# Histone methyltransferase G9a protects against acute liver injury through GSTP1

**DOI:** 10.1038/s41418-019-0412-8

**Published:** 2019-09-12

**Authors:** Yu Zhang, Weili Xue, Wenquan Zhang, Yangmian Yuan, Xiuqin Zhu, Qing Wang, Yujuan Wei, Dong Yang, Chen Yang, Yan Chen, Yu Sun, Shun Wang, Kun Huang, Ling Zheng

**Affiliations:** 1 Tongji School of Pharmacy, Tongji Medical College, Huazhong University of Science and Technology, Wuhan, 430030 Hubei PR China; 2 Hubei Key Laboratory of Cell Homeostasis, College of Life Sciences, Wuhan University, Wuhan, 430072 Hubei PR China; 3 Department of Blood Transfusion, Wuhan Hospital of Traditional and Western Medicine, Wuhan, 430022 PR China

**Keywords:** Epigenetics, Endocrine system and metabolic diseases

## Abstract

Acute liver injury is commonly caused by bacterial endotoxin/lipopolysaccharide (LPS), and by drug overdose such as acetaminophen (APAP). The exact role of epigenetic modification in acute liver injury remains elusive. Here, we investigated the role of histone methyltransferase G9a in LPS- or APAP overdose-induced acute liver injury. Under d-galactosamine sensitization, liver-specific G9a-deficient mice (L-G9a^−/−^) exhibited 100% mortality after LPS injection, while the control and L-G9a^+/−^ littermates showed very mild mortality. Moreover, abrogation of hepatic G9a or inhibiting the methyltransferase activity of G9a aggravated LPS-induced liver damage. Similarly, under sublethal APAP overdose, L-G9a^−/−^ mice displayed more severe liver injury. Mechanistically, ablation of G9a inhibited H3K9me1 levels at the promoters of *Gstp1/2*, two liver detoxifying enzymes, and consequently suppressed their transcription. Notably, treating L-G9a^−/−^ mice with recombinant mouse GSTP1 reversed the LPS- or APAP overdose-induced liver damage. Taken together, we identify a novel beneficial role of G9a-GSTP1 axis in protecting against acute liver injury.

## Introduction

As the main detoxifying organ, liver shows remarkable capacity against bacterial/viral infection and drug toxicity, thus playing a central role in the regulation of body homeostasis [1]. Acute liver injury is a serious health issue with high morbidity and mortality [2]. Bacterial lipopolysaccharide (LPS), also termed endotoxin, is known to cause acute liver injury and sepsis [3]. Drug toxicity is another leading cause of acute liver injury, overdose of nonprescription analgesic acetaminophen (APAP) accounts for more than 50% of all acute liver failure cases in USA [2].

LPS- and APAP overdose-induced acute liver failure share similar pathological mechanisms. Upon LPS stimulation, immune cells are recruited to the liver to produce proinflammatory cytokines, thus triggering inflammatory response; consequently, elevated inflammation-associated molecules are found in LPS-injured livers [3]. Similarly, a substantial number of neutrophils infiltrate the liver after APAP overdose [4]. Inflammation/reactive nitrogen species (RNS)/reactive oxygen species (ROS)-induced cell death also plays pathogenic roles in LPS/APAP-induced acute liver injury [4]. Pathological production of large amount of nitric oxide (NO) by inducible NO synthase (iNOS) leads to the formation of RNS, which further nitrates proteins to exacerbate inflammation and tissue injury [4]. Besides leading to hepatocyte death, ROS-caused oxidative stress also aggravates inflammatory responses through promoting the infiltration and activation of immune cells, and consequently contributes to the pathogenesis of LPS/APAP-induced acute liver injury [5].

G9a, a ubiquitously expressed histone methyltransferase encoded by *EHMT2* (euchromatic histone lysine N-methyltransferase 2), is a member of the SET domain-containing Su(var)3-9 family [6]. G9a and its related protein GLP (G9a-like protein, encoded by *EHMT1*) catalyze mono- and di-methylation on histone H3K9 (H3K9me1 and H3K9me2), which are respectively associated with transcriptional activation and repression. Currently, most functional studies focus on G9a-mediated H3K9me2, whereas the roles of G9a-mediated H3K9me1 are less reported [7]. G9a plays important roles in diverse cellular processes, including proliferation, differentiation, senescence, and replication [7]. However, the role of G9a in acute liver injury remains unclear.

Here, using liver-specific G9a-deficient (L-G9a^−/−^) mice, we found abrogation of G9a exacerbated LPS- or APAP overdose-induced liver injury through increasing immune cells infiltration and RNS/ROS production, aggravating DNA damage and hepatocyte death. We further identified that the downregulation of glutathione S-transferase Pi (GSTP), the liver detoxifying enzyme [8], was, at least, partially responsible for the exacerbated liver injury, since recombinant GSTP1 effectively suppressed the LPS- or APAP overdose-induced liver damage in L-G9a^−/−^ mice. These results suggest G9a-GSTP1 axis as a potential therapeutic target for acute liver injury and associated diseases.

## Materials and methods

### Animals

Male C57BL/6 mice were obtained from the Center for Animal Experiment/Animal Biosafety Level-III Laboratory of Wuhan University. Breeding pairs of B6.Cg-Tg (Alb-cre)21Mgn mice (Alb-Cre) were obtained from the Jackson Laboratory. Floxed G9a (G9a^flox/flox^) mice with C57BL/6 background were generated by the Model Animal Research Center of Nanjing University using standard protocols. Briefly, the targeting vector was designed to introduce a Neo cassette in intron 21 and LoxP sites in intron 7 and 21 of Ehmt2. Embryonic stem (ES) cells were electroporated with this vector and clones with correct insertion were identified by PCR and southern blot analyses. Correct ES cells were injected into C57BL/6 blastocysts to create chimeric mice, which were bred to obtain germ-line transmission of targeted G9a allele. G9a^flox/flox^ mice were first crossed with Alb-Cre mice, and the heterozygous offspring carrying Alb-Cre transgene (G9a^flox/wt^-Cre) were then intercrossed to generate homozygous mice carrying Alb-Cre transgene (G9a^flox/flox^-Cre), which were used as liver-specific G9a knockout (L-G9a^−/−^) mice. Other littermates, G9a^wt/wt^-Cre or mice carrying no Alb-Cre transgene were used as the controls (Con). Mice were housed in ventilated microisolator cages with free access to water and regular chow. Sample sizes, as described in figure legends, were selected based on effect size and availability as per usual standard. Sample sizes (*n*) indicated in figure legends refer to the number of animal. Randomization was done by selecting animals of similar age and weight. Blinding was involved in histological studies. Animals were handled according to the Guidelines of the China Animal Welfare Legislation, as approved by the Committee on Ethics in the Care and Use of Laboratory Animals of College of Life Sciences, Wuhan University.

### Genotyping

Genotyping was performed by PCR assay using genomic DNA obtained from mouse tail. The primer set 5′-TGTGAGTTCCAGGTAGTGGC-3′ and 5′-GAATGCCACACAGCAGTGAC-3′ were used to detect the wildtype allele (229 bp) and the G9a LoxP allele (347 bp) (Fig. S1A). Mice carrying Alb-Cre transgene were detected by the primer set 5′-GAACCTGATGGACATGTTCAGG-3′ and 5′-AGTGCGTTCGAACGCTAGAGCCTGT-3′.

### LPS, d-galactosamine (d-Gal), BIX01294 (BIX), and APAP injection

LPS (*E. coli*, 0111:B4) and d-Gal were purchased from Sigma (St Louis, MO), and APAP was obtained from Macklin (Shanghai, China). BIX, a specific G9a methyltransferase activity inhibitor [9], was obtained from Targetmol (Boston, MA). For d-Gal and LPS co-treatment, male mice were intraperitoneally (i.p.) injected with 200 mg/kg BW (body weight) of d-Gal and 3 mg/kg BW of LPS, or same volume of vehicle, and mortality was recorded within 36 h (Fig. S1B). Liver samples were collected either immediately from the dead mice, or from surviving mice at 36 h after co-injection. Blood samples were collected from tail vein of all surviving mice at 16 h after co-injection. For LPS-only injection, male and female mice were i.p. injected with a single dose of LPS (3 mg/kg BW) or same volume of vehicle (Fig. S1C). For BIX and LPS co-treatment, C57BL/6 male mice were i.p. injected with BIX (50 mg/kg BW) for 2 successive days. On day 2, LPS (3 mg/kg BW) was given after BIX injection, while mice injected with vehicle or BIX or LPS alone were used as the controls (Fig. S1D). For APAP overdose-induced acute liver injury, male mice were i.p. injected with APAP (400 mg/kg BW) or same volume of vehicle after overnight fasting (Fig. S1E). At 1 or 24 h after LPS/APAP injection, serum and livers were collected. Histological evaluation and western blots were performed at 24 h after LPS or APAP injection; while quantitative real-time PCR (qPCR) was performed at 1 or 24 h after injection.

### Cell culture, primary hepatocyte isolation, plasmid construction, transfection/infection, and BIX treatment

HepG2 (human hepatocellular carcinoma cell line) cells and Hepa1-6 (murine hepatoma cell line) cells, which were purchased from Procell (Wuhan, China) and Rochen (Shanghai, China), respectively. Cells were cultured at 37 °C in a 5% CO_2_ incubator, and maintained in DMEM (Hyclone, South Logan, UT, USA) supplemented with 10% FBS (Hyclone) and 1% penicillin–streptomycin (Hyclone). Primary mouse hepatocytes were isolated as previously reported [10]. In brief, liver was perfused with calcium‐free solution and then digested with collagenase (Sigma, St. Louis, MO) perfusion. Dispersed cells were released, and hepatocytes were collected and plated in collagen-coated plates with DMEM media plus 10% FBS. Plates were washed 4 h later to remove non-adherent cells, and the adherent hepatocytes were used for experiments.

Two short hairpin RNAs targeting human G9a sequence, shG9a-1 and shG9a-2 (5′-GGTTTGCGCTTCAACTCAA-3′ and 5′-GGCGATTGCTCCAGGAATT-3′, respectively), were cloned into the pSUPER plasmid. HepG2 cells were transfected with shScram (containing a scrambled nontargeting sequence) or shG9a plasmid by using TurboFect transfection reagent (Thermo Scientific, Rockford, IL), and stable knockdown cells were selected with 1 μg/μl puromycin (Amresco, Solon, OH).

Two systems were used to overexpress mouse G9a in primary hepatocytes. PAdeno-MCMV-EGFP-P2A-G9a-3*Flag (Ad-G9a) and control plasmids were constructed, packed into adenovirus, and purified (Obio, Shanghai, China). Primary hepatocytes were infected with G9a or control adenovirus at an MOI of 50 for 6 h. Alternatively, primary hepatocytes were transfected by pCAGGS-mG9a or control vector with lipofectamine 2000 (Invitrogen, Grand Island, NE). Cells was collected at 48 h after infection or transfection. For BIX (Cayman, Ann Arbor, MI) treatment, HepG2 cells or primary hepatocytes were treated with different dosages of BIX (0, 2, 4, or 8 μM) for 24 h. All the experiments were repeated at least three times with 2–3 samples per group at each time.

### Preparation and administration of recombinant GSTP1 protein (rGSTP1)

Mouse *Gstp1* cDNA was amplified by PCR and cloned into pET-28a, which contains an N-terminal His-tag. rGSTP1 was expressed in *E. Coli* and purified using Ni^2+^-NTA agarose (Qiagen, Valencia, CA) as previously described [11]. L-G9a^−/−^ mice were intravenously (i.v.) injected with rGSTP1 (10 mg/kg) or vehicle at 30 min prior to LPS or APAP injection, and sacrificed at 24 h after the injection (Fig. S1F–G).

### Western blots and quantitative real-time PCR (qPCR)

Tissues were sonicated in ice-cold RIPA buffer (Beyotime, China) and protein concentrations were determined. Total RNA was extracted using RNAiso Plus (TaKaRa Biotechnology, Japan). Targeted protein expression levels were quantitated relative to the internal control in the same sample, and then normalized to the respective control group, which was arbitrarily set as 1. qPCR was performed with *β-actin* as the internal control, with the relative difference of targeted gene expressed as fold change calculated by the 2^−ΔΔCT^ method. Antibodies and primers used were listed in Table S1.

### Protein identification by mass spectrometry (MS)

After SDS-PAGE and coomassie brilliant blue staining, gel pieces at target positions in each group were cut, subjected to in-gel digestion, and analyzed with a Q Exactive HF mass spectrometer coupled with an Easy-nLC 1000 system (Thermo Scientific, Rockford, IL). The MS data were processed using Thermo Proteome Discoverer software. MS spectra were searched by the SEQUEST algorithm against SwissProt database of mouse. The mass tolerances for precursor and fragment ions were set to 10 ppm and 0.02 Da, respectively. Search results were filtered to 1% false discovery rate using the target-decoy strategy on both peptide and protein levels.

### Serum alanine aminotransferase (ALT) and aspartate aminotransferase (AST) measurements

Serum ALT and AST levels were measured by an ADVIA 2400 Chemistry System (Siemens, Tarrytown, NY) with reagents purchased from DiaSys (DiaSys Diagnostic Systems, Shanghai, China) and Kehua (Kehua Bio-engineering, Shanghai, China), respectively.

### Determination of glutathione (GSH) level

Liver GSH levels were measured by a glutathione assay kit (Beyotime) following the manufacturer’s instructions. Absorbance at 412 nm was recorded and GSH levels were calculated from a standard curve.

### Histological analysis, immunochemistry staining, and TUNEL assay

Liver tissues were collected and routinely embedded into paraffin. Liver sections were stained with hematoxylin and eosin (H&E) or prepared for immunochemistry staining and TUNEL assay. For immunochemistry staining, after quenching the endogenous peroxidases with 3% H_2_O_2_ and blocking unspecific binding with 2% bovine serum albumin (Amresco, Solon, OH), sections were stained with F4/80, Ly-6G, CD3, 3-nitrotyrosine, 8-oxoG, or Nrf2 (Table S1) overnight at 4 °C. After extensive washing, sections were incubated with respective biotinylated secondary antibodies. Positive staining was visualized using DAB Substrate (Cwbiotech, Beijing, China) following the ABC Kit (Vector Laboratories, Burlingame, CA). For TUNEL assay, apoptotic cells were detected using a TUNEL Bright Green Apoptosis Detection Kit (Vazyme Biotech, Nanjing, China) according to the manufacturer’s instructions. Positive stained areas were quantified and positive cells were counted using the Image-Pro Plus software in 4–6 fields randomly selected for each sample.

### Chromatin immunoprecipitation (ChIP) assay

ChIP assay was performed as we previously described [12]. Briefly, liver tissues were minced and crosslinked with 1% formaldehyde, then quenched with 125 mM glycine. After washed with cold PBS, samples were resuspended with digestion buffer plus 1 mM PMSF. Crosslinked chromatin was sheared with Micrococcal Nuclease (New England Biolabs, Beverly, MA) and sonication. Chromatin was immunoprecipitated using anti-H3K9me1, anti-H3K9me2, and anti-G9a (all from Abcam, Cambridge, MA) or respective IgGs (Sigma, MO; or Santa Cruz, CA). The immune-complexes were captured with Protein G agarose beads (GE Healthcare, Chicago, IL). ChIP-enriched and input DNA were extracted and analyzed by qPCR, with the inputs as the internal control. Primers for ChIP assay were provided in Table S1. Regions of GSTP1/2 promoter used for ChIP assay were ranged from −2000 bp to TSS (transcription start site), which was downloaded from http://genome.ucsc.edu/cgi-bin/hgNear. This region was averagely divided into four parts to cover the promoter area by one set of primer per 500 bp, and the primers (Table S1) were randomly but not selectively designed by *Primer 5*.

### Detection of intracellular rGSTP1 by confocal microscopy

To detect the intracellular transport of the rGSTP1, we labeled rGSTP1 with fluorescein isothiocyanate (FITC) to treat the Hepa1-6 cells. Briefly, cells were starved for 12 h before being treated with or without inhibitors of endocytosis (chlorpromazine hydrochloride (CPZ; Targetmol, Washington, MA; 20 μM), dynasore (Targetmol; 50 μM) or MβCD (Targetmol; 0.5 mM)) for 1 h, followed by 1 h co-incubation with FITC or FITC-GSTP1 (1 μM). Images were taken under a ZEISS 780 confocal microscope (ZEISS, Oberkochen, Germany).

### Statistical analysis

The data were expressed as average ± standard deviation (SD). Statistical significance was determined by analyzing the data with the nonparametric Kruskal–Wallis test followed by the Mann–Whitney test for comparison of three or more than three groups, or with the Mann–Whitney test only for comparison of two groups. Differences were considered statistically significant at *p* < 0.05.

## Results

### Hepatic G9a deletion is fatal upon LPS/d-Gal injection

**Fig. 1:**
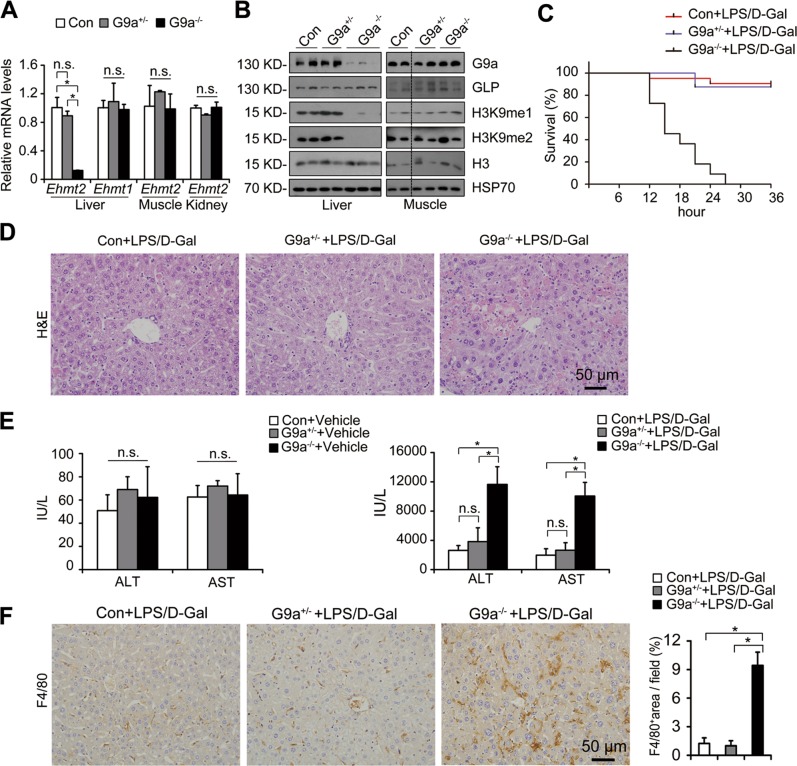
Hepatic G9a deletion aggravates LPS-induced liver damage. **a** Relative mRNA levels of *Ehmt2* and *Ehmt1* in the liver, muscle and kidney of Control, L-G9a^+/−^ and L-G9a^−/−^ mice. **b** Representative western blots for G9a, GLP, H3K9me1, H3K9me2, H3, and HSP70 in the liver and muscle of Control, L-G9a^+/−^ and L-G9a^−/−^ mice. **c** Survival rate (*n* = 21 for Controls; *n* = 16 for L-G9a^+/−^ mice; *n* = 11 for L-G9a^−/−^ mice) and **d** H&E staining (*n* = 6–8 per group; scale bar, 50 μm) after LPS/d-Gal co-injection. **e** Serum levels of ALT and AST with or without LPS/d-Gal co-injection. **f** Representative pictures for F4/80 staining in livers *(left)* and quantitative results *(right)* after LPS/d-Gal co-injection (scale bar, 50 μm). All data were obtained from male mice. *n* = 3–4 per group; n.s., not significant; ^*^p < 0.05

Significantly downregulated *Ehmt2*/G9a and H3K9me1/me2 levels, but not *Ehmt1*/GLP, were observed in livers of liver-specific G9a-deficient (L-G9a^−/−^) mice (Fig. 1a, b). No significant difference was found on *Ehmt2*/G9a and H3K9me1/me2 levels between the livers of heterozygous L-G9a^+/−^ and control mice (Fig. 1a, b), possibly due to the random monoallelic gene expression [13]. Meanwhile, the abundance of *Ehmt2*/G9a, *Ehmt1*/GLP, and H3K9me1/me2 was similar in the muscle of control, L-G9a^+/−^ and L-G9a^−/−^ mice (Fig. 1a, b). Moreover, the levels of GLP were not significantly altered in shG9a stable HepG2 cells (Fig. S2A, B). Contrary to the embryonic lethal phenotype of whole-body G9a knockout mice [14], L-G9a^−/−^ mice were viable and fertile with no obvious abnormality.

LPS/d-Gal co-injection is a classical way to induce acute liver injury, in which d-Gal increases the sensitivity of rodents to LPS-induced hepatotoxicity [15]. All LPS/d-Gal co-treated L-G9a^−/−^ mice died within 27 h, whereas the L-G9a^+/−^ and control mice showed very mild mortality with no significant difference between two groups within 36 h (Fig. 1c). H&E staining was conducted, compared with the L-G9a^+/−^ and control mice, the livers of L-G9a^−/−^ mice exhibited severe hemorrhage, swollen hepatocytes and larger injured area (Fig. 1d). Moreover, serum ALT and AST levels, two clinical parameters of liver injury, were significantly increased in L-G9a^−/−^ mice compared with those of the L-G9a^+/−^ and control mice under LPS/d-Gal co-injection, while the levels were similar among three groups without stress (Fig. 1e). More severe F4/80 staining was found in livers of L-G9a^−/−^ mice compared with those of the L-G9a^+/−^ and control mice under LPS/d-Gal co-injection (Fig. 1f). These results indicated that under d-Gal sensitization, LPS induced a fatal acute liver injury in L-G9a^−/−^ mice. Since L-G9a^+/−^ mice showed comparable responses to LPS/d-Gal as the controls, only L-G9a^−/−^ and control mice were used in following studies.

### Hepatic G9a deletion augments LPS-induced immune cells infiltration and RNS/ROS production

To study the role of G9a in LPS-induced acute liver injury while avoiding significantly mouse death, LPS was used alone. Consistently, histological examination revealed more aggravated immune cells infiltration in livers of LPS-injected L-G9a^−/−^ males compared with the controls, which was absent in vehicle-injected groups (Fig. S3).

**Fig. 2:**
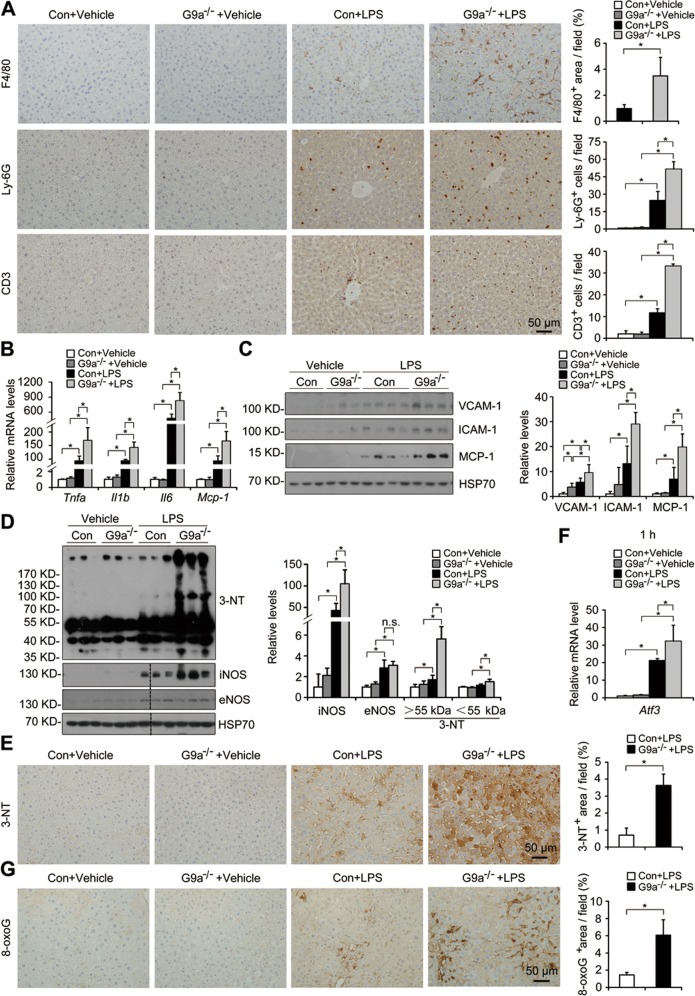
Hepatic G9a deletion augments LPS-induced inflammation, RNS/ROS production, DNA damage and hepatocyte apoptosis. **a** Representative pictures for F4/80, Ly-6G and CD3 staining in the liver *(left)* and quantitative results *(right)*. **b** qPCR results of the indicated genes in the liver of different groups. **c, d** Representative western blots *(left)* and quantitative results *(right)* for VCAM-1, ICAM-1, and MCP-1 (**c**), iNOS, eNOS and 3-NT (**d**) in livers of different groups. HSP70 serves as the loading control. **e** Representative pictures for 3-NT staining in the liver *(left)* and quantitative results *(right)*. **f** Relative mRNA level of *Atf3* in the liver at 1 h after LPS injection. **g** Representative pictures for 8-oxoG staining in the liver *(left)* and quantitative results *(right)*. *n* = 3–4 per group; 3-NT, 3-Nitrotyrosine; n.s., not significant; ^*^p < 0.05; scale bar, 50 μm

LPS induces liver injury mainly through inflammation. After LPS injection, increased F4/80 (marker for macrophages), Ly-6G (marker for neutrophils), and CD3 (marker for T cells) staining were detected in livers of the control males, while further increased infiltration of these immune cells were found in L-G9a^−/−^ males (Fig. 2a). Consistently, at 1 h after LPS injection, *Tnfa*, *Il1b*, *Il6*, and monocyte chemotactic protein 1 (*Mcp-1*) were significantly elevated in the control livers, and further elevated in livers of L-G9a^−/−^ males (Fig. 2b). Adhesion molecules, such as vascular cell adhesion molecule-1 (VCAM-1) and intercellular adhesion molecule-1 (ICAM-1), recruit leukocytes to injury sites to initiate inflammatory responses. After LPS injection, increased levels of VCAM-1, ICAM-1, and MCP-1 were found in livers of the control males, while their levels were further elevated in L-G9a^−/−^ males (Fig. 2c). Similar results were found in females (Fig. S4A–C).

RNS production augments inflammation and tissue injury [16]. After LPS injection, increased iNOS and eNOS levels were detected in livers of male controls, and further dramatically increased iNOS but not eNOS level was detected in L-G9a^−/−^ males (Fig. 2d). 3-nitrotyrosine (3-NT) is a “molecular fingerprint” indicating the formation of nitrated proteins [17]. After LPS injection, significantly elevated nitrated proteins were found in livers of L-G9a^−/−^ males compared with the controls, despite of the similar levels of NOSs and nitrated proteins in these two groups under physiological conditions (Fig. 2d, e).

To test whether oxidative damage is involved in the exacerbated LPS-induced liver injury of L-G9a^−/−^ mice, we measured the levels of *Atf3* and 8-oxoguanine (8-oxoG), a ROS-induced transcription factor and a marker for oxidative damage, respectively [4]. Without LPS injection, no difference was found in livers of male L-G9a^−/−^ and their controls (Fig. 2f, g). In livers of LPS-injected male controls, elevated *Atf3* and 8-oxoG staining were found; which were further elevated in LPS-injected L-G9a^−/−^ males (Fig. 2f, g). Similar results were seen in females (Fig. S4D–G).

### Hepatic G9a deletion augments DNA damage and hepatocyte apoptosis upon LPS injection

**Fig. 3:**
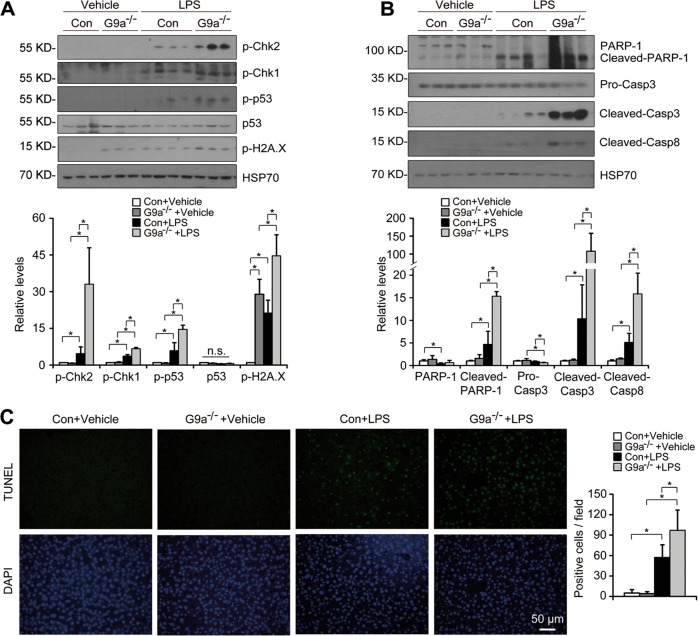
Hepatic G9a deletion augments DNA damage and hepatocyte apoptosis upon LPS injection. **a, b** Representative western blots *(up)* and quantitative results *(down)* for markers of DNA damage response signaling (**a**) and apoptosis (**b**) in livers of different groups. HSP70 serves as the loading control. **c** Representative pictures for TUNEL staining in the liver *(left)* and quantitative results *(right)*. All data were obtained from male mice. *n* = 3–4 per group; n.s., not significant; ^*^p < 0.05; scale bar, 50 μm

Mounting evidences suggest that nitrative and oxidative stresses induce DNA damage [18]. After LPS challenge, the levels of p-Chk2, p-Chk1, p-p53 and p-H2A.X were dramatically increased in the controls, and were further increased in livers of L-G9a^−/−^ males; whereas the p53 level was similar in these groups (Fig. 3a). Hepatocyte apoptosis plays a pathogenic role in acute liver injury [18]. Without LPS challenge, the levels of apoptosis-associated proteins and the number of TUNEL^+^ cells were similar in livers of L-G9a^−/−^ males and their controls (Fig. 3b, c). After LPS injection, increased levels of apoptosis-related cleaved fragment of poly(ADP-ribose) polymerase-1 (PARP-1), cleaved Caspase3/8, and increased number of TUNEL^+^ cells were found in livers of the controls; and these markers were further upregulated in livers of LPS-injected L-G9a^−/−^ males (Fig. 3b, c). Similar results were found in females (Fig. S5A–C).

### Inhibiting G9a methyltransferase activity exacerbates LPS-induced liver damage

**Fig. 4:**
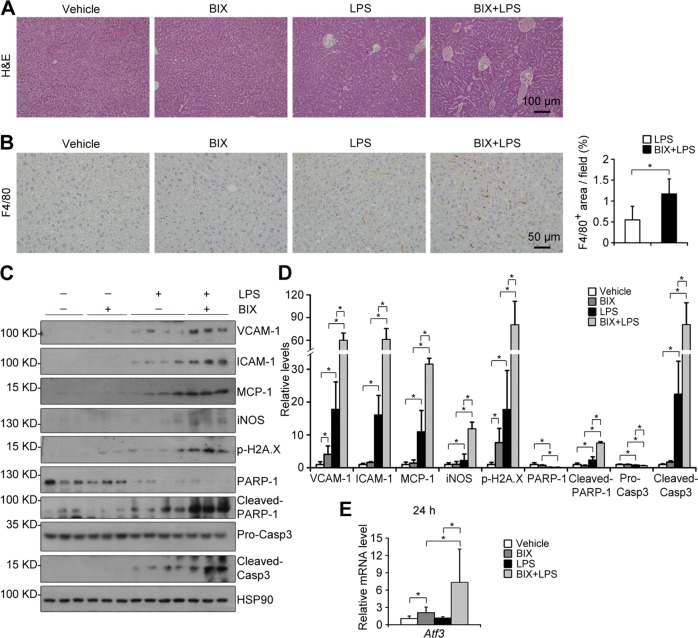
Inhibiting G9a methyltransferase activity deteriorates LPS-induced liver damage. **a, b** Representative pictures for H&E staining (**a**; scale bar, 100 μm); and F4/80 staining (**b***, left*; scale bar, 50 μm) with quantitative results (**b***, right)* in the liver. **c, d** Representative western blots (**c**) and quantitative results (**d**) of indicated proteins in livers of different groups. HSP90 serves as the loading control. **e** Relative mRNA level of *Atf3* in the liver at 24 h after LPS injection. *n* = 3–4 per group; ^*^p < 0.05

To investigate whether G9a-deficiency enhanced acute liver damage depends on its methyltransferase activity, BIX01294 (BIX), an inhibitor for the methyltransferase activity of G9a, was used [9]. The specificity of BIX was first evaluated. In WT primary hepatocytes and HepG2 cells, BIX significantly downregulated H3K9me1/me2 levels (Fig. S6A-B). In G9a^−/−^ primary hepatocytes, downregulated H3K9me1/me2 levels were found as expected, however, no further downregulation of H3K9me1/me2 levels was observed after BIX treatment (Fig. S6C). Consistently, BIX could not further downregulate hepatic H3K9me1/me2 levels in L-G9a^−/−^ males (Fig. S6D). H&E and F4/80 staining suggested BIX *per se* did not cause significant pathological changes; in contrast, BIX markedly increased accumulation of macrophages in the liver upon LPS injection (Fig. 4a, b). Consistently, inflammatory molecules such as VCAM-1, ICAM-1, MCP-1, and iNOS, DNA damage marker p-H2A.X, as well as apoptosis markers like cleaved PARP-1 and Caspase3, were significantly increased in livers of BIX and LPS co-treated males, compared with those treated with either one (Fig. 4c, d). Moreover, compared with LPS-treated males, further elevated hepatic *Atf3* was detected in co-treated males (Fig. 4e). These results suggest a methyltransferase activity dependent role of G9a on liver injury.

### Deletion of G9a exacerbates APAP-induced acute liver injury

**Fig. 5:**
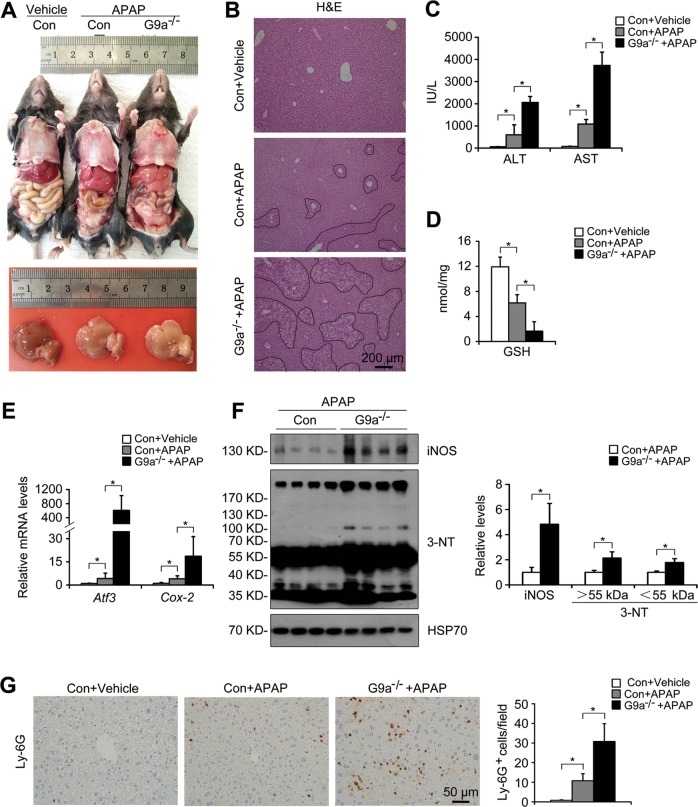
Deletion of G9a exacerbates APAP overdose-induced acute liver injury. **a** Representative pictures of livers from different groups at 24 h after APAP injection. **b** Representative pictures for H&E staining in the liver; scale bar, 200 μm. **c** Serum levels of ALT and AST. **d** Levels of GSH in the liver. **e** Relative mRNA levels of *Atf3* and *Cox-2* in the liver. **f** Representative western blots *(left)* and quantitative results *(right)* for the indicated proteins in the liver of different groups. HSP70 serves as the loading control. **g** Representative pictures for Ly-6G staining in the liver *(left)* and quantitative results *(right)*; scale bar, 50 μm. *n* = 4 per group; ^*^p < 0.05

To further verify the role of G9a on acute liver injury, an APAP overdose model was used. At 24 h after a sublethal APAP overdose, L-G9a^−/−^ males displayed deteriorated physical states compared with the controls (Movie S1). In the controls, APAP overdose caused massive hepatic toxicity as indicated by gross injured liver (Fig. 5a), typical centrilobular necrosis demonstrated by H&E staining (Fig. 5b), and increased serum ALT/AST levels (Fig. 5c). Under APAP stress, L-G9a^−/−^ males displayed more severe liver necrosis and much higher serum ALT/AST (Fig. 5a–c). The toxicity of APAP comes from its metabolite N-acetyl benzoquinoneimine, which triggers hepatic damage by depleting glutathione (GSH) [19]. APAP overdose significantly decreased hepatic GSH level of the control males, while more pronounced decrease was observed in L-G9a^−/−^ males (Fig. 5d), as well as aggravated oxidative stress indicated by elevated liver *Atf3* and *cyclooxygenase-2* (*Cox-2*) levels of L-G9a^−/−^ males (Fig. 5e). APAP overdose also dramatically upregulated the hepatic iNOS and 3-NT in livers of L-G9a^−/−^ males compared with the controls (Fig. 5f). Meanwhile, after APAP overdose, increased Ly-6G staining was detected in the control livers, while further increased neutrophils infiltration was found in L-G9a^−/−^ mice (Fig. 5g).

### Loss of G9a represses *Gstp1/2*

**Fig. 6:**
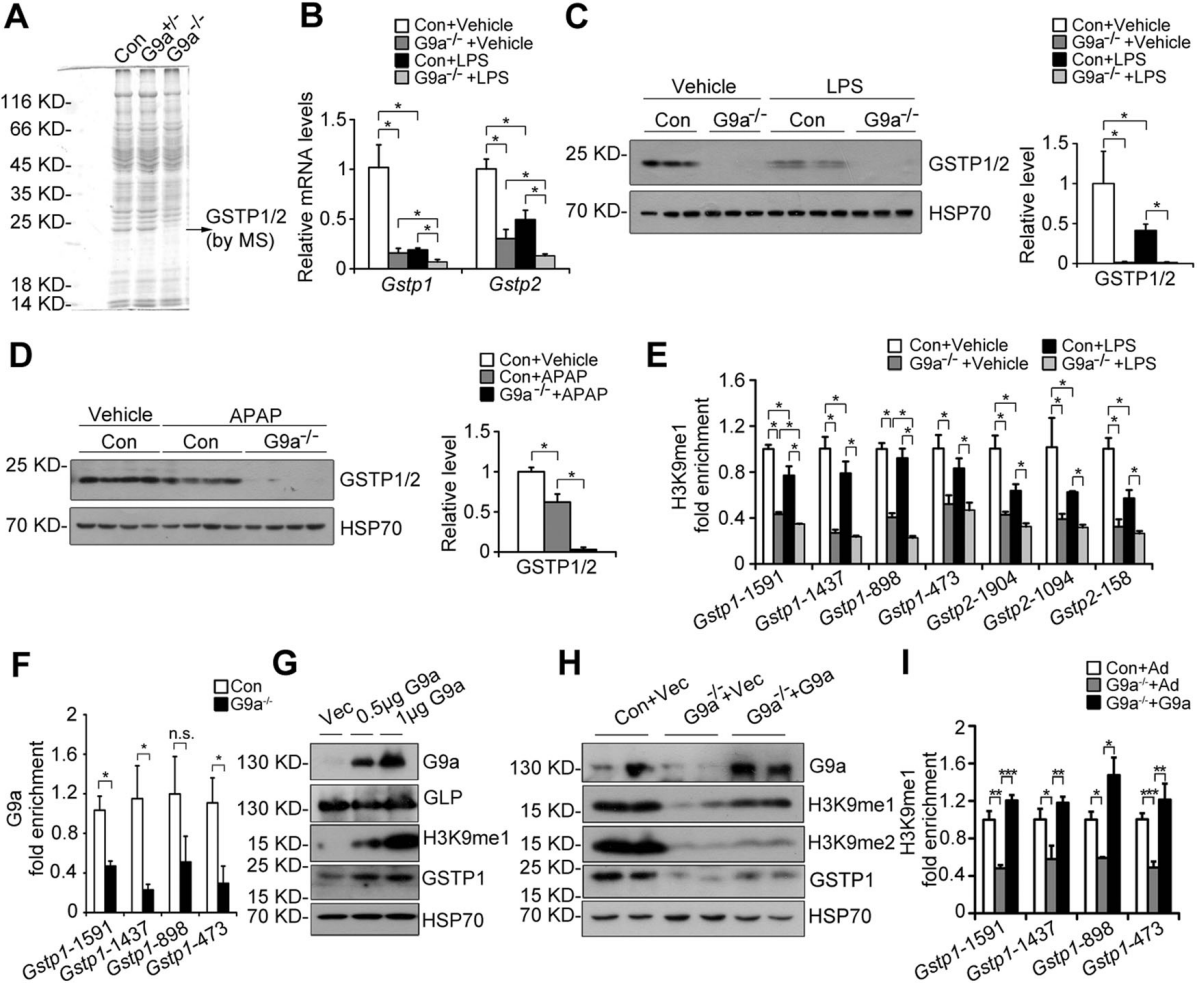
G9a mediates H3K9me1 at the promoters of *Gstp*. **a** Coomassie brilliant blue staining of whole liver extracts from Control, L-G9a^+/−^ and L-G9a^−/−^ mice. **b** Relative mRNA levels of *Gstp1* and *Gstp2* in the liver of different groups at 24 h after LPS injection. **c, d** Representative western blots of GSTP1/2 *(left)* and quantitative results *(right)* in livers of different groups. HSP70 serves as the loading control. **e** ChIP-qPCR analysis of H3K9me1 levels at the promoters of *Gstp1* and *Gstp2* in livers of different groups at 24 h after LPS injection. **f** ChIP-qPCR analysis of G9a levels at the promoter of *Gstp1* in livers of control and L-G9a^−/−^ mice (error bars indicate SEM). Above data (**a**–**f**) were obtained from mice with *n* = 3–4 per group. **g, h** Representative western blots of GSTP1/2, G9a, H3K9me1, H3K9me2, or GLP after overexpression of pCAGGS-mG9a in primary hepatocytes obtained from WT (**g**) or G9a^−/−^ (**h**). HSP70 serves as the loading control. Similar results were observed from the Ad-G9a transfection. **i** ChIP-qPCR analysis of H3K9me1 levels at the promoters of *Gstp1* in control, G9a^−/−^ or adenovirus-mediated G9a transfected G9a^−/−^ primary hepatocytes. ^*^p < 0.05

Compared with the controls, Coomassie brilliant blue staining of whole liver lysate indicated a faint band in L-G9a^−/−^ males about 23 kDa with or without LPS challenge (Figs. 6a, S7), further MS study identified two top candidates, GSTP1 and GSTP2 (Fig. S8). GSTP is an isoform of glutathione S-transferases (GSTs), which are a family of phase II detoxifying enzymes. Mouse has two GSTP genes, *Gstp1* and *Gstp2* [20]. We found *Gstp1* is much more abundant than *Gstp2* in mouse liver (Fig. S9A). The levels of GSTP1/2 were significantly decreased in livers of L-G9a^−/−^ mice compared with the controls, with or without LPS challenge; and LPS dramatically downregulated GSTP1/2 levels in the controls (Figs. 6b, c, S9B). Consistently, in a recent study, genes of six different pathways were screened by real-time PCR in L-G9a^−/−^ mice, and *GSTP1* is one of the more than 80 altered genes screened with a real-time PCR approach [21]. Furthermore, BIX treatment decreased GSTP1/2 levels with or without LPS stress (Fig. S9C), indicating a G9a methyltransferase activity dependent regulation of GSTP1/2. Consistently, APAP overdose downregulated GSTP1/2 levels moderately in the controls and drastically in L-G9a^−/−^ mice (Fig. 6d). Since GSTPs not only play roles in protecting cells against toxins (drugs and carcinogens) and oxidative stress, but also have anti-inflammation and anti-apoptosis effects [20, 22], we thus hypothesize that the downregulation of GSTP1/2 in livers of L-G9a^−/−^ mice contributes to the exacerbated liver injury upon LPS or APAP overdose stress.

### G9a mediates H3K9me1 at the promoters of *Gstp1/2*

We next investigated the mechanisms by which G9a regulates *Gstp1/2*. Transcription factor Nrf2 (NFE-2 related factor 2) regulates expression of GSTPs [23]. Under normal condition, Nrf2 binds to Keap1 (Kelch-like ECH-associated protein 1) in cytoplasm. Upon oxidative stress, the Nrf2-Keap1 complex is disrupted, the released Nrf2 translocates to the nucleus and activates targeted genes [24]. We tested whether G9a deficiency inhibits *Gstp1/2* expression via repressing the Keap1-Nrf2 axis. We found no differences on the total levels of Nrf2 and Keap1 in livers of the controls and L-G9a^−/−^ males with or without LPS injection; however, LPS did induce significant downregulation of total Nrf2 and Keap1 levels in these two groups (Fig. S9D, E). Furthermore, similar nuclear Nrf2 staining were observed in livers of the controls and L-G9a^−/−^ males; whereas LPS-treatment induced similar reduction of total and nuclear Nrf2 in livers of both groups (Fig. S9F). Consistently, the levels of multiple Nrf2 targeted genes, including *Hmox1*, *Nqo1*, *Gcl*, and *Mrp1*, were similar in livers of the control and L-G9a^−/−^ mice, with or without LPS injection (Fig. S9G). Thus, the alteration of GSTP1 in L-G9a^−/−^ mice may not due to changes in Nrf2 signaling.

G9a regulates gene expression by adding repressive marker H3K9me2 or transactivating marker H3K9me1 at gene promoters [9], thus we hypothesize that G9a may directly regulate *Gstp1/2* through epigenetic modification. ChIP assay indicated strong enrichment of H3K9me1, but no H3K9me2, in the promoter regions (−2000 bp to TSS, transcription start site) of *Gstp1/2* in livers of wildtype mice (Fig. S10). In the controls, the H3K9me1 levels were mildly repressed in some promoter regions of *Gstp1/2* upon LPS injection (Fig. 6e); whereas G9a-deficiency significantly reduced the H3K9me1 levels at the promoters of *Gstp1/2* with or without LPS stress, and the H3K9me1 levels were further decreased in some promoter regions of *Gstp1* in livers of L-G9a^−/−^ mice upon LPS injection (Fig. 6e). In the controls, G9a bound to the same promoter regions of *Gstp1*, and G9a-deficiency significantly reduced binding (Fig. 6f). However, decreased H3K9me2 levels at the promoters of inflammatory genes, such as *Tnfa*, *Il1b*, and *Il6*, were seen in livers of L-G9a^−/−^ mice upon LPS injection (Fig. S11).

Next, we overexpressed G9a in wildtype primary hepatocytes. Increased levels of H3K9me1 and GSTP1/2, but not GLP, were observed (Fig. 6g). Re-constitution of G9a in L-G9a^−/−^ primary hepatocytes rescued downregulated H3K9me1/me2 and GSTP1/2 (Fig. 6h); meanwhile, ChIP assay demonstrated rescued H3K9me1 binding to the promoter of *Gstp1* (Fig. 6i).

### rGSTP1 inhibits the aggravated liver injury induced by LPS or APAP overdose in G9a-dificient mice

**Fig. 7:**
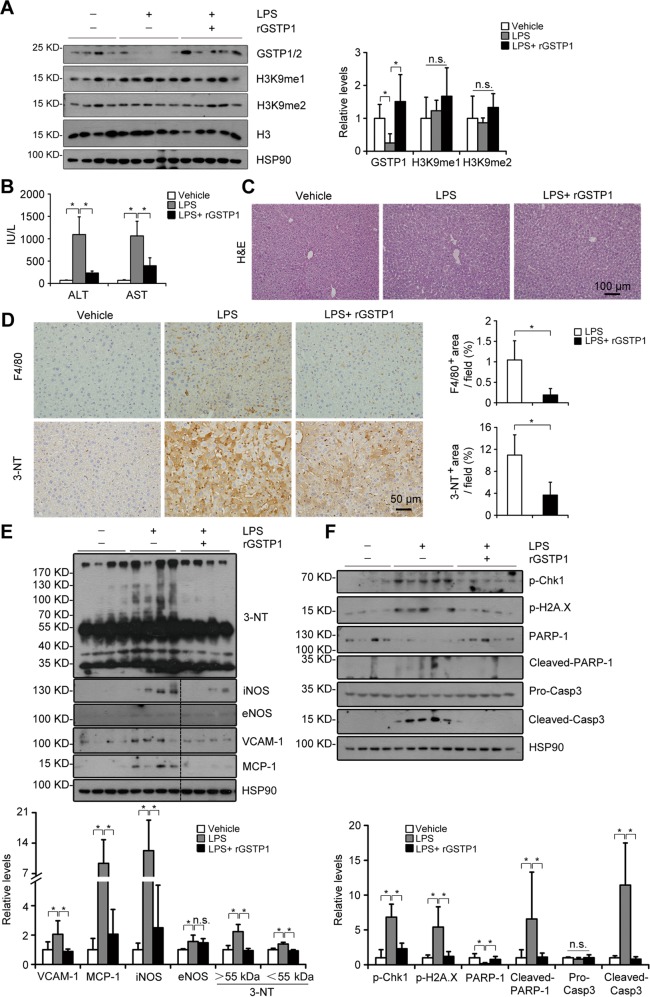
rGSTP1 attenuates LPS-induced liver damage in L-G9a^−/−^ mice. **a** Representative western blots *(left)* and quantitative results *(right)* for GSTP1/2, H3K9me1/me2 in the livers of different groups. H3 or HSP90 serves as the loading control. **b** Serum levels of ALT and AST. **c** Representative pictures for H&E staining in the liver; scale bar, 100 μm. **d** Representative pictures for F4/80 and 3-NT staining *(left)* and quantitative results *(right)*; scale bar, 50 μm. **e, f** Representative western blots *(up)* and quantitative results *(down)* of the indicated proteins in livers of different groups. HSP90 serves as the loading control. All these data were obtained from male L-G9a^−/−^ mice. *n* = 4–6 per group; ^*^p < 0.05

To address whether the protective effects of G9a on LPS- or APAP-induced liver damage is mediated by GSTPs, we administered rGSTP1 to L-G9a^−/−^ mice. Exogenous rGSTP1 effectively rescued the LPS-induced GSTP1/2 reduction in the livers without significantly affecting H3K9me1/me2 levels (Fig. 7a). Under LPS injection, rGSTP1-treated mice showed better physical activity (Movie S2), as well as significantly lower serum ALT/AST levels (Fig. 7b). rGSTP1 also significantly inhibited LPS-induced accumulation of macrophages and nitrated proteins, elevation of VCAM-1, MCP-1, iNOS, and 3-NT levels, and upregulated some antioxidant genes such as *Sod2*, *Cat*, *Trx1,* and *Gpx2*, in livers of L-G9a^−/−^ mice (Fig. 7c–e, S12A). Furthermore, rGSTP1 significantly inhibited DNA damage and hepatocyte apoptosis induced by LPS in livers of L-G9a^−/−^ mice (Fig. 7f).

**Fig. 8:**
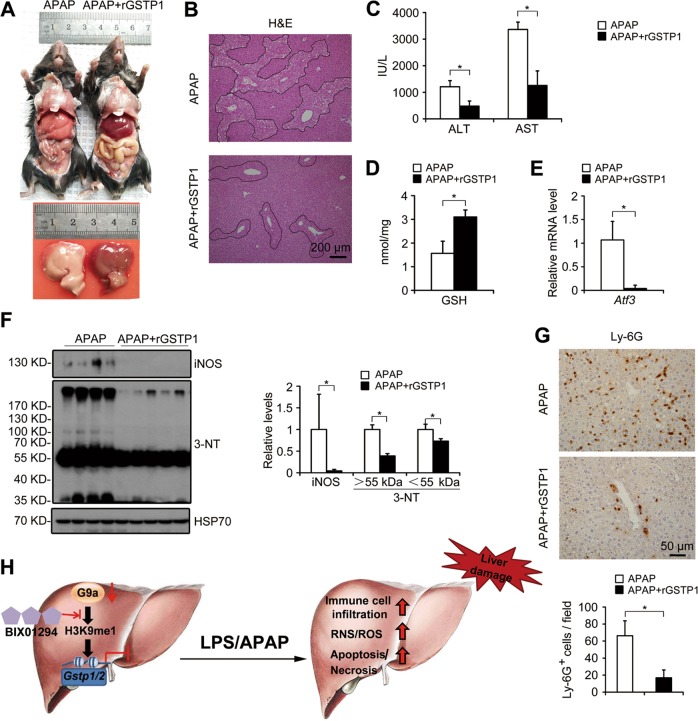
rGSTP1 attenuates APAP-induced liver damage in L-G9a^−/−^ mice. **a** Representative pictures of livers from different groups at 24 h after APAP injection. **b** Representative pictures for H&E staining in the liver; scale bar, 200 μm. **c** Serum levels of ALT and AST. **d** Levels of GSH in the liver. **e** Relative mRNA level of *Atf3* in the liver. **f** Representative western blots *(left)* and quantitative results *(right)* for the indicated proteins in livers of different groups. HSP70 serves as the loading control. **g** Representative pictures for Ly-6G staining in the liver *(up)* and quantitative results *(down)*; scale bar, 50 μm. All these data were obtained from male L-G9a^−/−^ mice. *n* = 4–6 per group; n.s., not significant; ^*^p < 0.05. **h** Working model for how G9a regulates acute liver injury

Similarly, at 24 h post APAP overdose, rGSTP1-treated L-G9a^−/−^ mice also showed better behavior and physical activity (Movie S3), alleviated hepatic toxicity (Fig. 8a) and centrilobular necrosis (Fig. 8b), as well as lower serum ALT/AST levels (Fig. 8c). Moreover, as an important detoxifying protein, rGSTP1 prevented L-G9a^−/−^ mice from GSH depletion (Fig. 8d), and oxidative damages as indicated by the lower *Atf3* level (Fig. 8e), as well as potentiated some antioxidant genes (Fig. S12B). Furthermore, rGSTP1 also significantly inhibited APAP-induced nitrated proteins accumulation in livers of L-G9a^−/−^ mice (Fig. 8f). Ly-6G staining suggested significantly decreased neutrophils infiltration in livers of rGSTP1-injected L-G9a^−/−^ mice compared with the controls (Fig. 8g). Together, these results demonstrate that rGSTP1 protects LPS- or APAP overdose-induced acute liver injury in L-G9a^−/−^ mice.

Whether rGSTP1 can be transported into cells was examined. FITC-labeled rGSTP1, but not free FITC, could be uptaken by Hepa1-6 cells (Fig. S13A), which was consistent with a previous report that rGSTP1 can be transported into cardiac tissue [22]. Moreover, transported rGSTP1 was markedly inhibited by CPZ or dynasore, two inhibitors for the clathrin-dependent endocytosis, but not by MβCD, an inhibitor for the caveolae-dependent endocytosis (Fig. S13B).

## Discussion

Acute liver injury, which has extremely poor prognosis and high mortality, lacks effective therapies. Here, we found liver-specific abrogation of G9a lead to aggravated acute liver injury upon LPS challenge in both genders. Mechanically, G9a upregulates GSTP1/2 levels, the liver detoxifying enzyme. Using two rodent models, we demonstrated that rGSTP1 treatment significantly rescued the acute liver injury in the L-G9a^−/−^ mice, based on liver function (indicated by ALT/AST levels), greater than 60% rescue effects were achieved by rGSTP1 treatment in both models at the dosage used (Figs. 7b, 8c).

H3K9me1 and H3K9me2 are generally regarded as epigenetic markers for gene activation and repression respectively, therefore altering G9a level may lead to changes of significant amount of genes. Consistently, previous reports have suggested that among differentially expressed genes, a portion (28–50%) are positively regulated by G9a [9]. Interestingly, although G9a regulates both H3K9me1 and H3K9me2 levels, most studies have been focused on the role of H3K9me2. Our results suggested that G9a regulates *Gstp1* through maintaining H3K9me1 levels at its promoter in the liver and cultured primary hepatocytes (Fig. 6e–i). It has been demonstrated that G9a positively regulates the transcription of *PHGDH* and *PSAT1*, two rate-limiting enzymes for *de novo* serine biosynthesis, through regulating H3K9me1 levels [9]. Similarly, BIX also prevents renal fibrosis by downregulating *klotho* with decreased H3K9me1 [25].

Our results showed globally affected levels of H3K9me1 and H3K9me2 by G9a-deficiency, but only H3K9me1 seemed to be regulated on *Gstp1* promoter (−2000 bp to TSS). This is not unusual for epigenetic regulations. In *Drosophila*, an enzyme activity impaired mutation of Trr, an H3K4me1 methyltransferase, significantly altered H3K4me1 levels; however, H3K4me1 levels were unchanged in some genes [26]. Furthermore, ChIP-seq revealed significantly changed H3K9me1 but not H3K9me2 levels on *PHGDH* and *SHMT2* gene loci after manipulating G9a level [9]; compared with the amount of H3K9me1 binding, H3K9me2 binding on *SHMT2* gene loci are much less [9]. Another report showed H3K9me2 but not H3K9me1 enrichment on the promoter or 5′-upstream region of some target genes of G9a, such as *Mege-a2* or *Wfdc15a* [27]. However, questions like how many types of modifications are simultaneously involved in regulating a specific gene promoter region, and the exact roles these specific enrichments in combination play, remain unclear.

The role of H3K9me2 on gene regulation may also contribute to protective effects of G9a on acute liver injury. Ablation or pharmacological suppression of G9a inhibits H3K9me2 on the promoter of *Beclin-1*, triggers the accumulation of intracellular ROS and activates NF-κB pathway in MCF-7 cells [28]. Furthermore, G9a methyltransferase-dependent regulation of H3K9me2 on the promoter of inflammatory genes, such as *TNFα* in THP-1 monocyte and *IFN-α/β* in MEFs, has been reported [29, 30]. Consistently, here we reported in vivo, that genetically or pharmacologically inhibiting G9a caused aggravated inflammation and ROS production in acute liver injury, which may in part due to the repression of H3K9me2 enrichment on the promoters of *Tnfa, Il1b, and Il6* (Figs. 2, 4, 5e–g, S4 and S11).

Besides function as a histone methyltransferase, G9a also can act in a histone methylation-independent but enzymatic activity dependent manner. By methylating estrogen receptor α, G9a can work as co-activator of nuclear receptors [31]. Whether G9a also works independent of histone methylation in acute liver injury awaits further investigation.

Notably, rGSTP1 treatment significantly inhibited LPS- or APAP-induced immune cells infiltration, RNS/ROS production, hepatocyte death and liver injury in L-G9a^−/−^ mice (Figs. 7, 8), suggesting that the aggravated liver damage induced by G9a deficiency was mediated, at least in part, by rGSTP1. Previous studies have shown that extracellular GSTP1 can cross plasma membrane, and rGSTP1 protects against infarction-induced heart failure and LPS-induced acute lung injury by reducing infarct area, apoptosis, and inflammation [22, 32]. Here, we also demonstrated that rGSTP1 can be transported into cells, possibly via clathrin-dependent endocytosis (Fig. S13). Despite of drastically reduced GSTP1/2 levels in liver, L-G9a^−/−^ mice exhibited no obvious physical defect under normal conditions; consistently, pathological investigation on liver reveals no significant abnormality [33]. However, GSTP^−/−^ mice are more susceptible to stress-induced organ injuries, including urinary bladder, skin and heart, exhibiting more aggravated inflammation, oxidative stress, apoptosis and consequently higher mortality [34–36]. GSTP1 catalyzes S-glutathionylation of a variety of highly genotoxic and cell-damaging molecules, and facilitates a variety of redox genes expression. Here, we found dramatically increased glutathione level, upregulation of several antioxidant genes, and decreased levels of iNOS and 3-NT, after rGSTP1 treatment (Fig. 8d–f, S12), suggesting the detoxification and redox function of rGSTP1 contribute to its hepatic protective effects.

In summary, our results indicate that G9a is essential in protecting against acute liver injury (Fig. 8h). Liver specific G9a-deficient mice show greater immune cells infiltration, RNS/ROS production and cell death upon endotoxin or APAP stimulation. G9a-regulated H3K9me1 at the promoters of *Gstp1/2* is responsible for the pathological and biochemical changes in endotoxin- or APAP-injected L-G9a^−/−^ mice, and rGSTP1 is necessary and sufficient to attenuate liver injury in L-G9a^−/−^ mice. Our study thus uncovers an epigenetic regulatory role of G9a in acute liver injury, and provides a molecular explanation for the beneficial role of G9a-GSTP1 axis against such injuries.

## Supplementary information

SUPPLEMENTAL MATERIAL

Supplementary video 1

Supplementary Video 2

Supplementary Video 3
