# Supplementary material for: Histone methyltransferase G9a protects against acute liver injury through GSTP1
Source: Cell Death Differ. 2019 Sep 12;27(4):1243–58. doi: 10.1038/s41418-019-0412-8 (PMC7206029; doi:10.1038/s41418-019-0412-8)
Supplement: Supplementary file 1 — SUPPLEMENTAL MATERIAL [file 41418_2019_412_MOESM1_ESM.pdf]

Supplementary Materials

Histone methyltransferase G9a protects against acute liver injury through GSTP1

Short title: G9a-GSTP1/2 axis in acute liver injury

Yu Zhang[1†], Weili Xue[2†], Wenquan Zhang[2], Yangmian Yuan[2], Xiuqin Zhu[2], Qing Wang[2], Yujuan Wei[1], Dong Yang[1], Chen Yang[1], Yan Chen[1], Yu Sun[2], Shun Wang[3], Kun Huang[1]* and Ling Zheng[2]*

[1]Tongji School of Pharmacy, Tongji Medical College, Huazhong University of Science and Technology, Wuhan, Hubei, P. R. China, 430030

[2]Hubei Key Laboratory of Cell Homeostasis, College of Life Sciences, Wuhan University, Wuhan, Hubei, P. R. China, 430072

[3]Department of Blood Transfusion, Wuhan Hospital of Traditional and Western Medicine, Wuhan, China, 430022

† These two authors contributed equally to this work.

* Corresponding author. Email: lzheng@whu.edu.cn (L.Z.); kunhuang@hust.edu.cn (K.H.)

This supplementary information contains thirteen figures, one table and three videos.

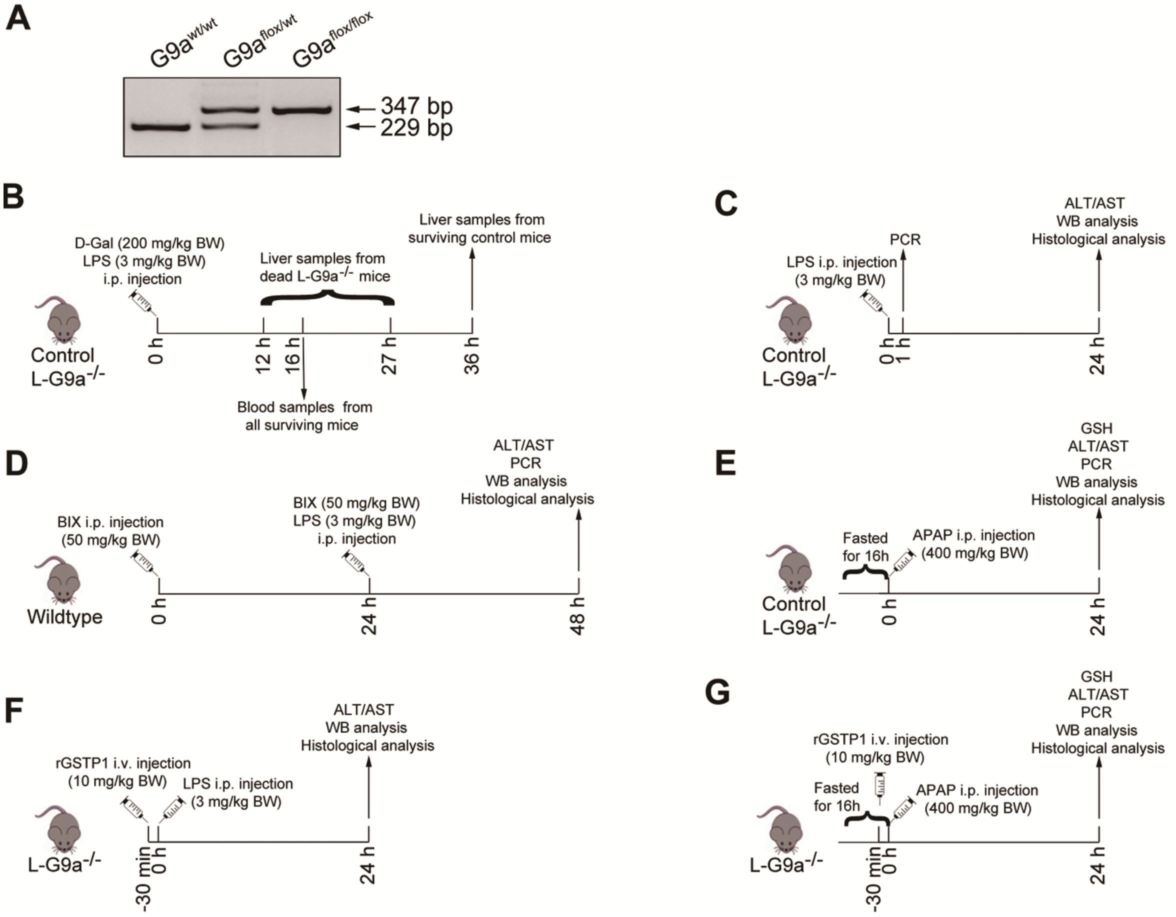

Fig. S1. Experimental design. (**A**) Genotype analysis by using genomic DNA extracted from the tails to demonstrated three genotypes of mouse. (**B-G**) Experimental design flowcharts of LPS/D-Gal-induced acute liver injury *(B)*, LPS-induced acute liver injury *(C)*, BIX and LPS co-treatment *(D)*, APAP-induced acute liver injury *(E)*, rGSTP1 treatment for L-G9a$^{-/-}$ mice under LPS injection *(F)* and APAP injection *(G)*. BW, body weight.

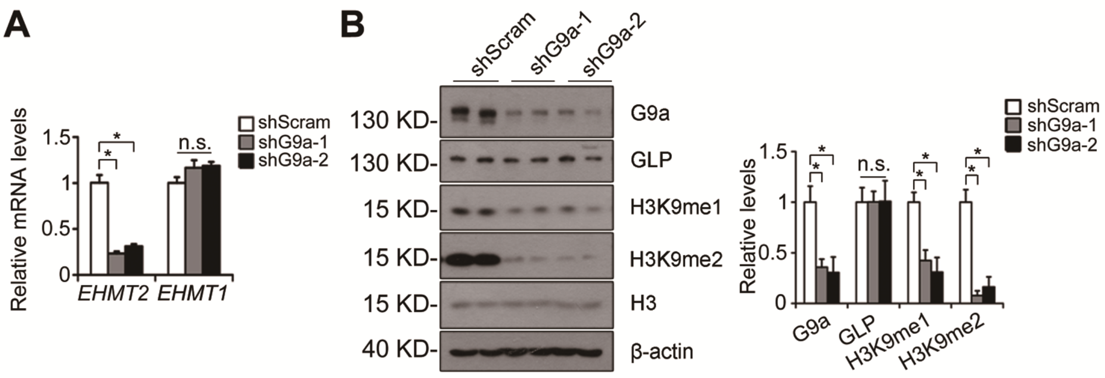

Fig. S2. Downregulation of G9a does not affect the expression level of GLP in HepG2 cells and in the livers after LPS treatment. (A) Relative mRNA level of *EHMT1/2* in control or two different shG9a stable HepG2 cell lines. **(B)** Representative western blots *(left)* and the quantitative results *(right)* for G9a, GLP and H3K9me1/me2 in the control or two different shG9a stable HepG2 cells. H3 and β-actin serve as the loading control. n=6; $^{*}p < 0.05$.

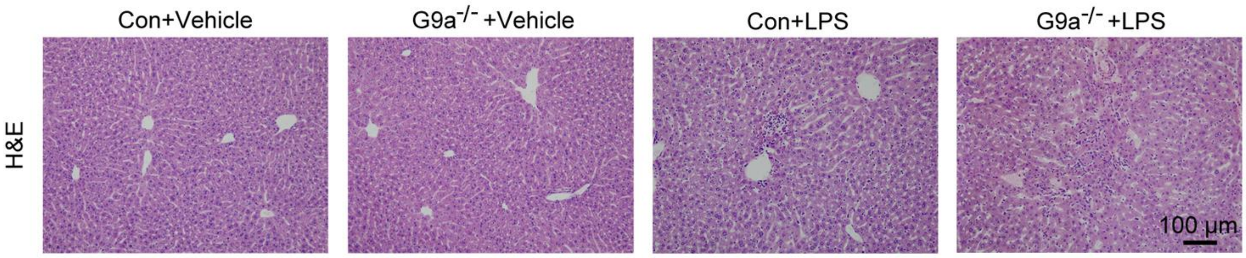

Fig. S3. Hepatic G9a deletion aggravates LPS-induced liver damage in male mice. H&E staining in livers after LPS injection. n=3-4 per group; scale bar, 100 µm.

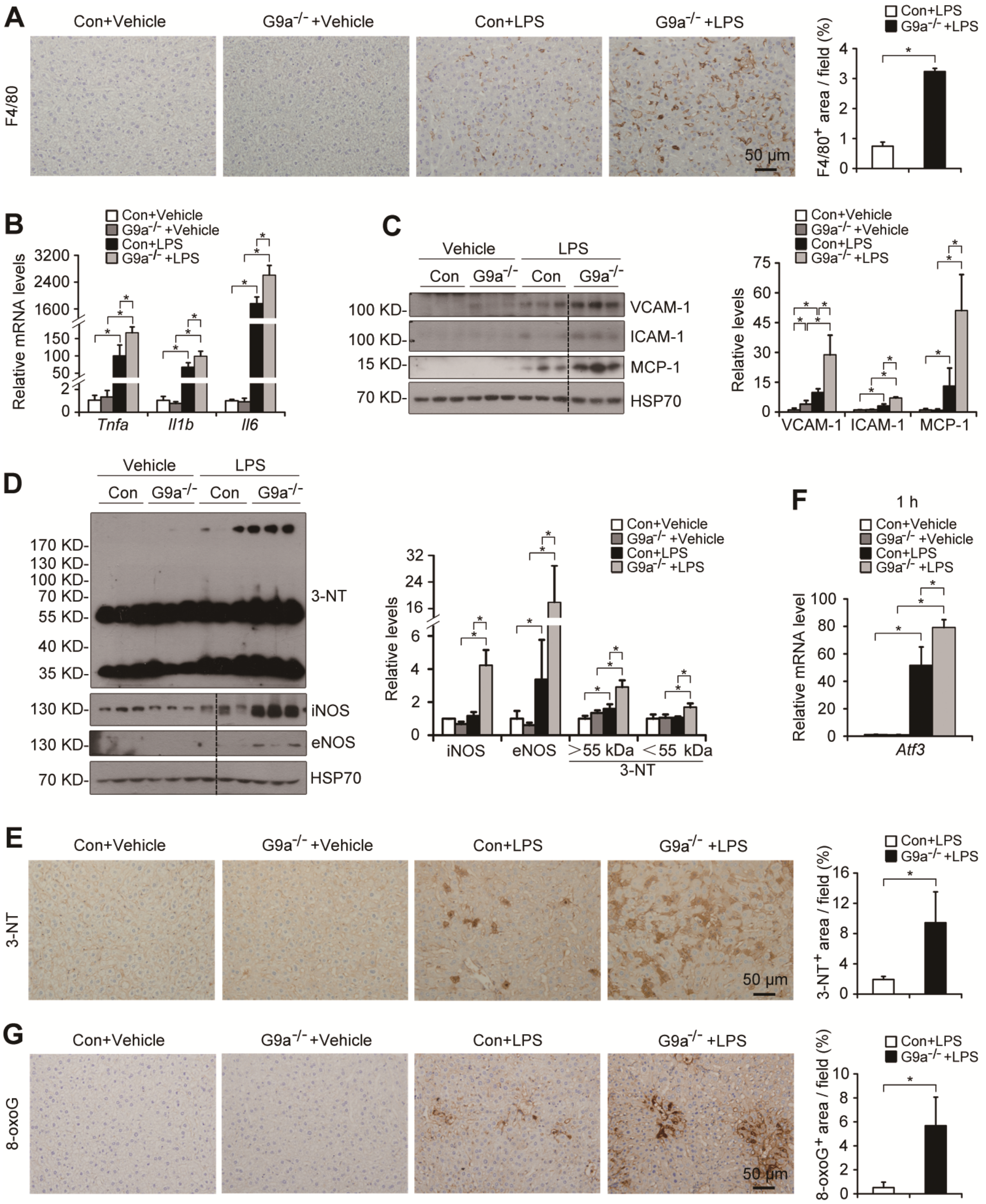

Fig. S4. Hepatic G9a deletion augments LPS-induced inflammation and RNS/ROS production in female mice. (A) Representative pictures for F4/80 staining in livers *(left)* and quantitative results *(right)*. (B) qPCR results of the indicated genes in livers of different groups. (C-D) Representative western blots *(left)* and quantitative results *(right)* for VCAM-1, ICAM-1 and MCP-1 *(C)*, iNOS, eNOS and 3-NT *(D)* in livers of different groups. HSP70 serves as the loading control. (E) Representative

pictures for 3-NT staining in livers *(left)* and quantitative results *(right)*. (F) Relative mRNA level of *Atf3* in the livers at 1 hour after LPS injection. (G) Representative pictures for 8-oxoG staining in livers *(left)* and quantitative results *(right).* n=3-4 per group; 3-NT, 3-Nitrotyrosine; *$p < 0.05$; scale bar, 50 μm.

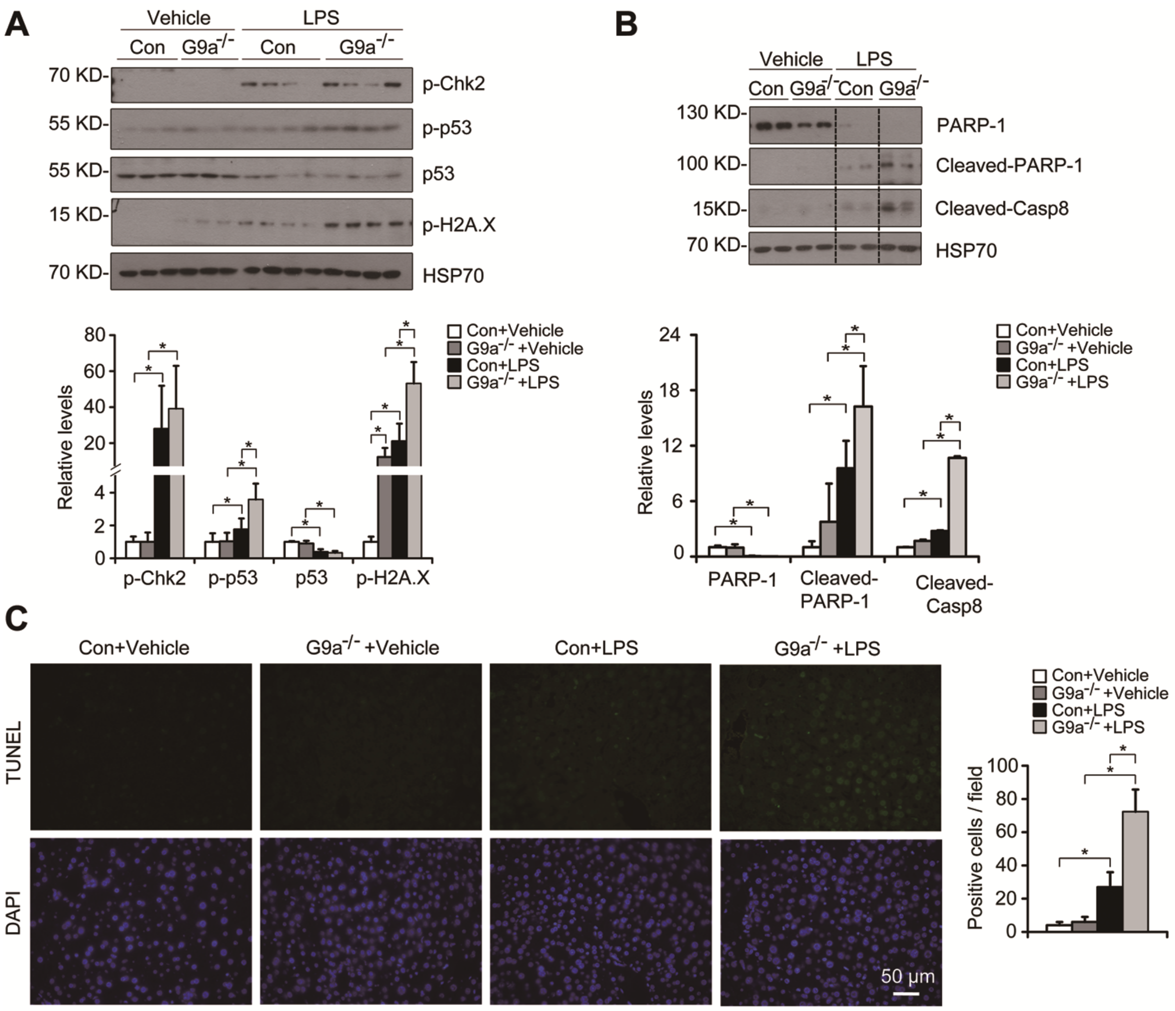

Fig. S5. Hepatic G9a deletion augments DNA damage and hepatocyte apoptosis upon LPS injection in female mice. (A-B) Representative western blots *(up)* and quantitative results *(down)* for markers of DNA damage response signaling *(A)* and apoptosis *(B)* in livers of different groups. HSP70 serves as the loading control. (C) Representative pictures for TUNEL staining in the livers *(left)* and quantitative results *(right)*. n=3-4 per group; **p* < 0.05; scale bar, 50 μm.

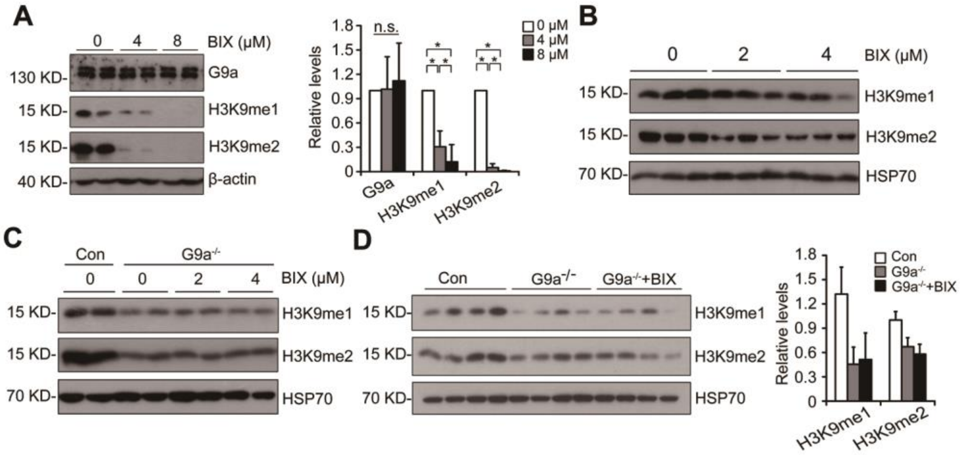

Fig. S6. BIX01294 (BIX) does not further inhibit H3K9me1/me2 levels in the G9a-deficient primary hepatocytes or livers. (A) Representative western blots *(left)* and quantitative results *(right)* for G9a, H3K9me1/me2 in HepG2 cells with indicated dosages of BIX. **(B-C)** Representative western blots for H3K9me1/me2 in primary hepatocytes isolated from the control mice *(B)*, as well as the control and L-G9a$^{-/-}$ mice *(C)* treated with indicated dosages of BIX. **(D)** Representative western blots for H3K9me1/me2 in livers of the control mice, L-G9a$^{-/-}$ mice and L-G9a$^{-/-}$ mice treated with BIX. β-actin or HSP70 serves as the loading control. n=6; n.s., not significant; $^{*}p < 0.05$.

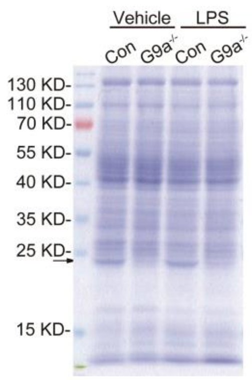

Fig. S7. The protein expression profile in the liver of different groups. Coomassie brilliant blue staining of whole liver extracts from control and L-G9a$^{-/-}$ mice with or without LPS i.p. injection. n=4 per group.

A

Name	MW (kDa)	Wildtype Coverage	Wildtype Unique Pepides	Wildtype PSMs	L-G9a$^{-/-}$ Coverage	L-G9a$^{-/-}$ Unique Pepides	L-G9a$^{-/-}$ PSMs	ΔPSMs
GSTP1	23.6	62.86	8	884	59.05	8	523	361
GSTP2	23.5	48.57	2	398	44.76	1	273	125
GSTA4	25.5	30.18	8	51	30.18	8	35	16
MGST1	17.5	18.06	2	22	7.74	1	9	13
CATB	37.3	13.57	5	30	15.93	5	20	10
SPRE	27.9	22.99	5	10	3.83	1	1	9

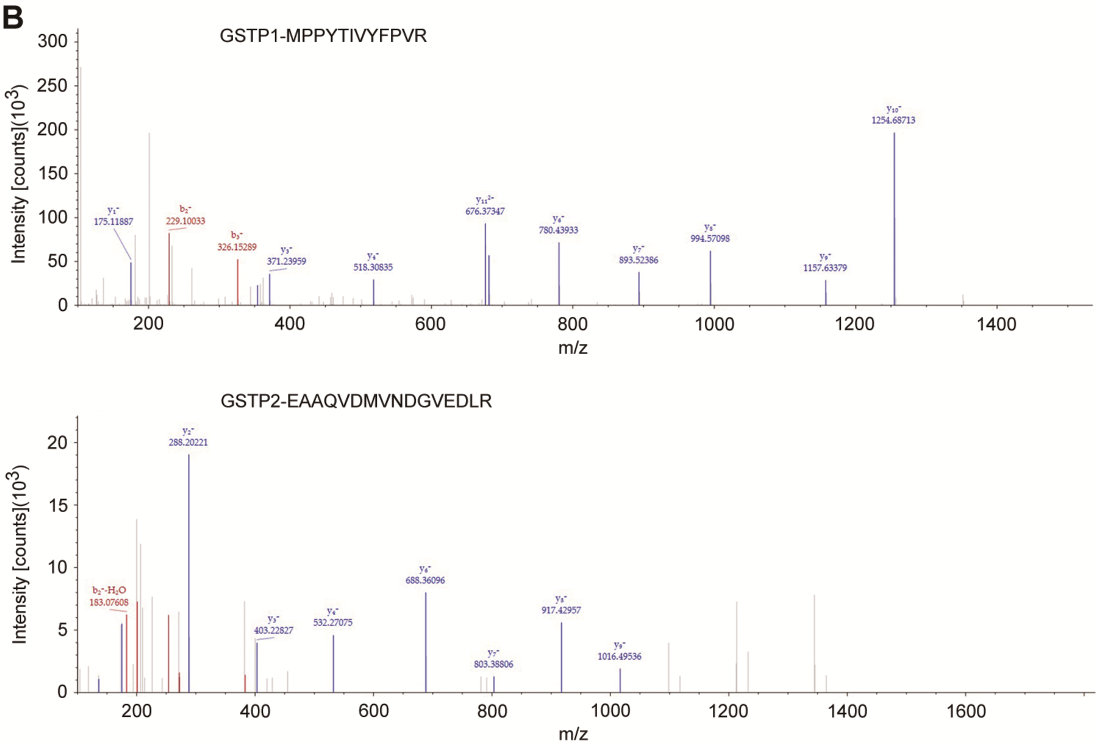

Fig. S8. Identification of GSTP1 and GSTP2 by mass spectrometry. (**A**) Top 6 candidate proteins by analyzing ΔPSM (wildtype − L-G9a$^{-/-}$). (**B**) Unique peptides of GSTP1 and GSTP2 identified by MS. GSTA4, glutathione S-transferase A4; MGST1, microsomal glutathione S-transferase 1; CATB, cathepsin B; SPRE, sepiapterin reductase.

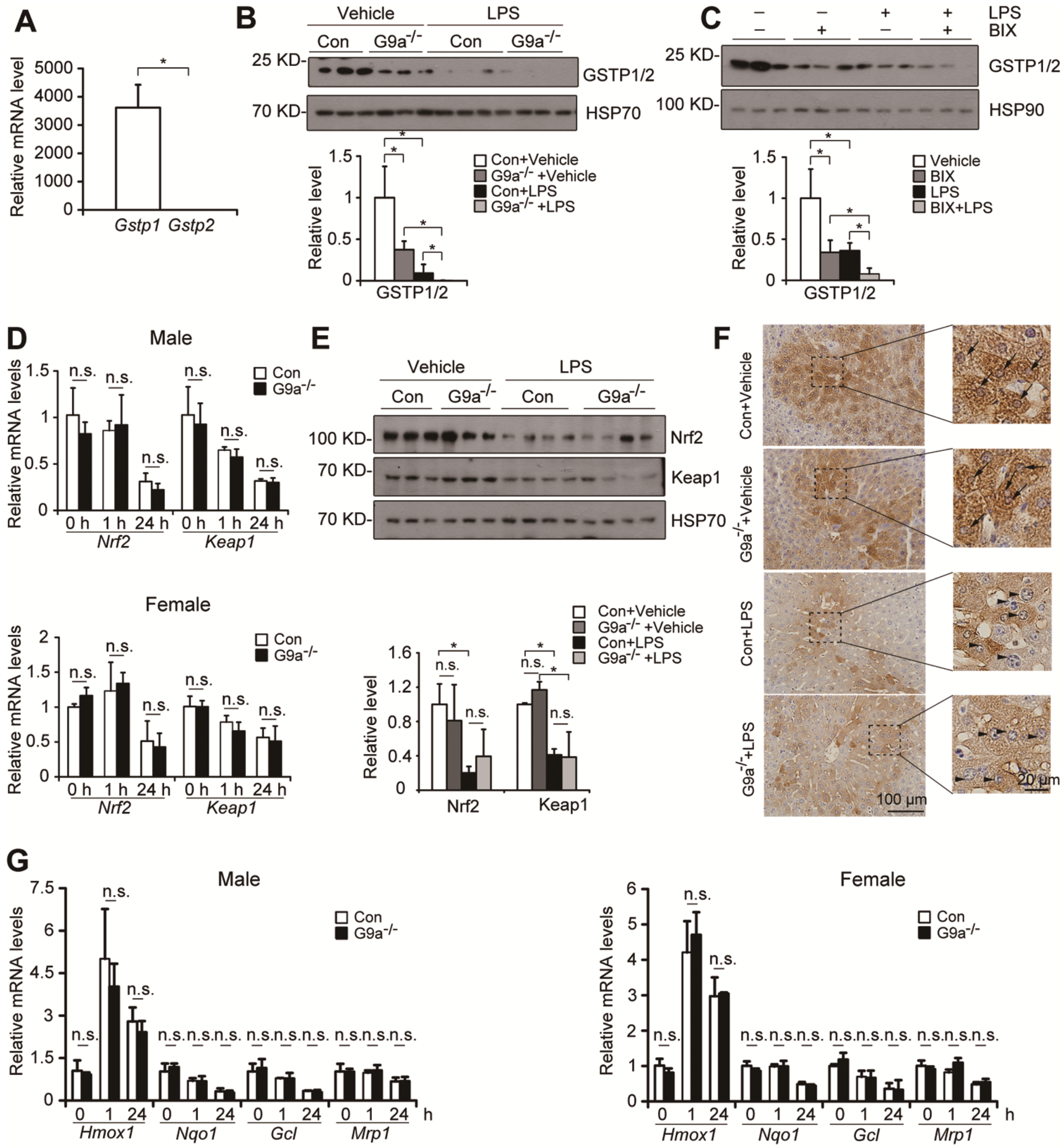

Fig. S9. G9a-deficiency-caused downregulation of GSTP1/2 is not regulated by Nrf2. (**A**) Relative mRNA levels of *Gstp1* and *Gstp2* in livers of the wildtype male mice. (**B-C**) Representative western blots *(up)* and quantitative results *(down)* for GSTP1/2 in livers upon LPS injury in female mice *(B)* and BIX with LPS co-treatment in male mice *(C)*. (**D**) Relative mRNA levels of *Nrf2* and *Keap1* in livers of the male *(up)* and female *(down)* control and L-G9a$^{-/-}$ mice at 0, 1 or 24 hours after LPS injection. (**E**)

Representative western blots *(up)* and quantitative results *(down)* for Nrf2 and Keap1 in livers of the control and L-G9a$^{-/-}$ male mice at 24 hours after LPS injection. (**F**) Representative pictures of Nrf2 staining in the livers of different groups. Nucleus with or without Nrf2 staining were marked by arrows or arrowheads, respectively. **(G)** Relative mRNA levels of *Hmox1*, *Nqo1*, *Gcl* and *Mrp1* in livers of the control and L-G9a$^{-/-}$ mice at 0, 1 or 24 hours after LPS injection. HSP70 or HSP90 serves as the loading control. n=3-4 per group; n.s., not significant; *$p < 0.05$.

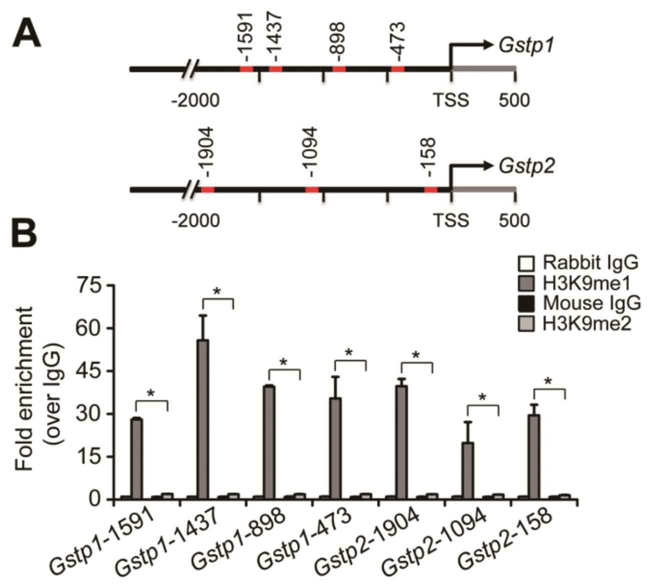

Fig. S10. H3K9me1/me2 levels at promoters of *Gstp1/2* in the livers of wildtype mice. (A) Locations of primers used for ChIP-qPCR. **(B)** ChIP-qPCR analysis of H3K9me1 and H3K9me2 levels at the various regions of promoters of *Gstp1* and *Gstp2* in livers of control mice. n=3 per group; **p* < 0.05.

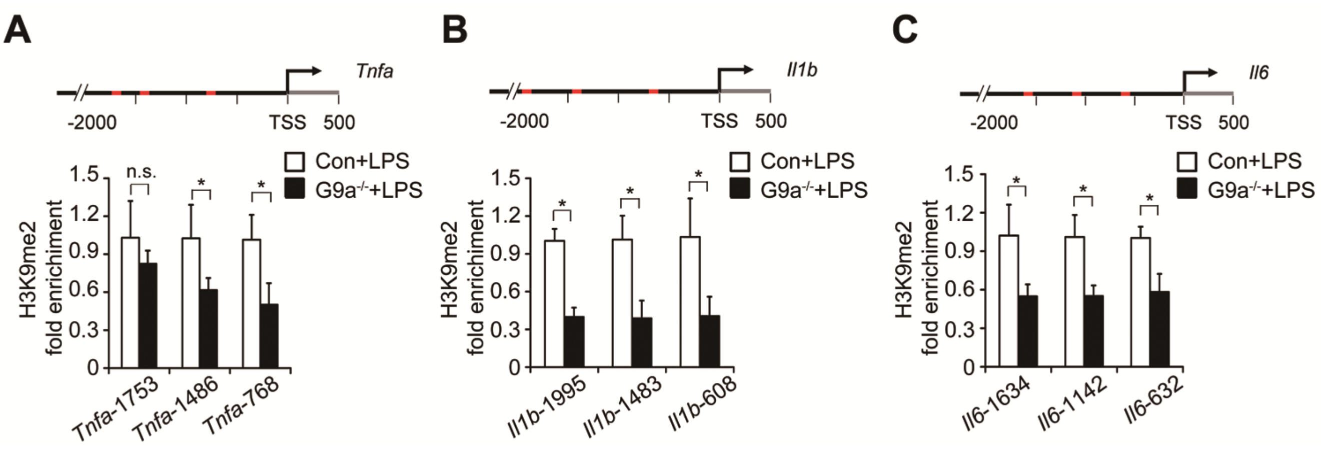

Fig. S11. H3K9me2 levels on the promoters of inflammatory genes in the livers of the control and L-G9a$^{-/-}$ mice upon LPS injection. (A-C) Locations of primers used for ChIP-qPCR *(up)* and the ChIP-qPCR analysis of the H3K9me2 at various promoter regions of inflammatory genes *(down)* such as *Tnfa (A)*, *Il1b (B)* and *Il6 (C)*. n=4 per group; n.s., not significant; *$p < 0.05$.

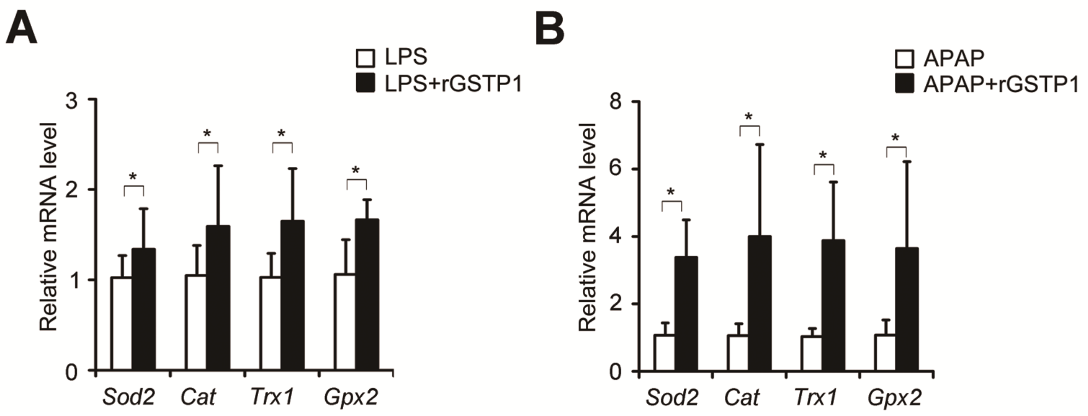

Fig. S12. rGSTP1 upregulates redox and detoxification genes in L-G9a$^{-/-}$ mice upon LPS- or APAP- induced liver damage. (**A-B**) Relative mRNA level of some redox and detoxification genes in the livers of indicated groups upon LPS- *(A)* or APAP- *(B)* stress. All these data were obtained from male L-G9a$^{-/-}$ mice. n=4-6 per group; $^{*}p < 0.05$.

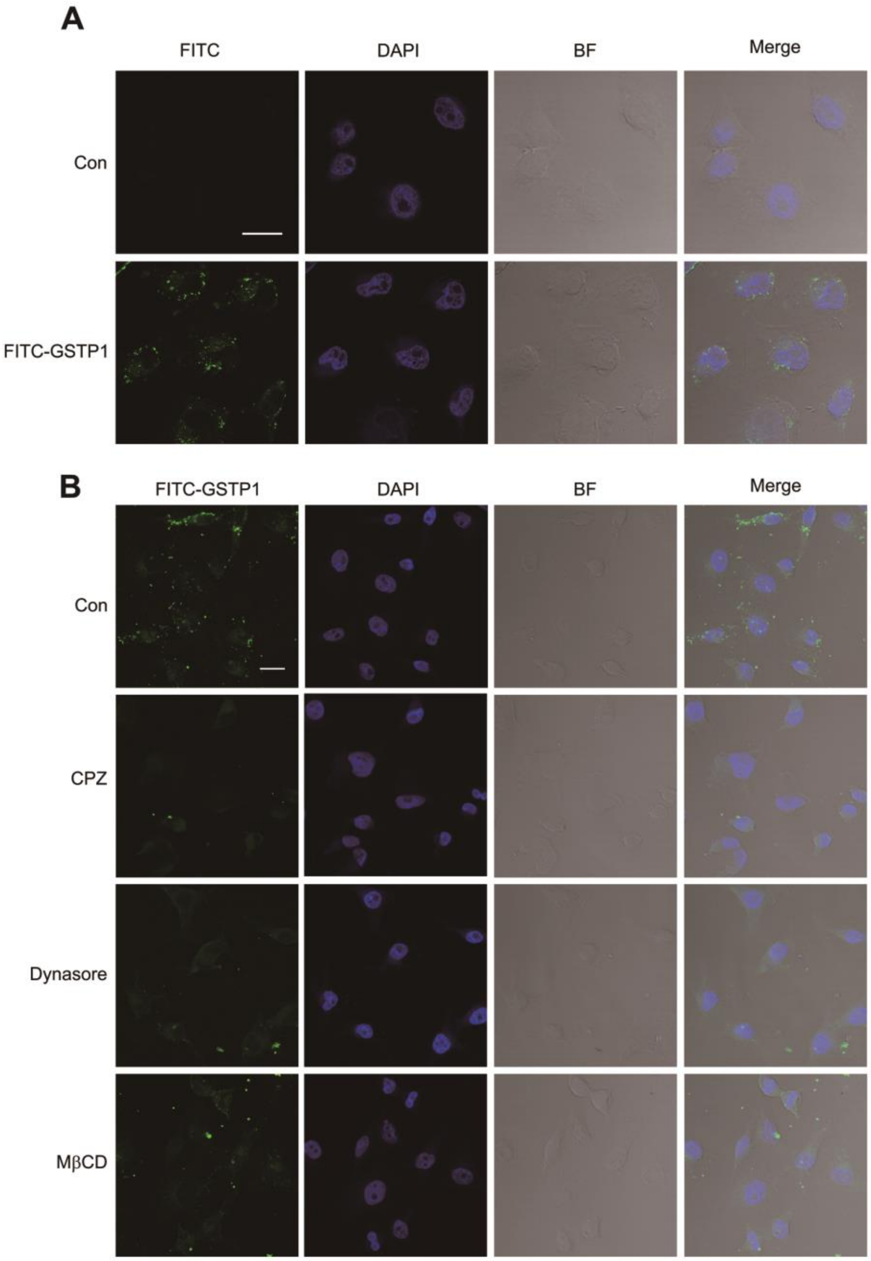

Fig. S13. rGSTP1 may be transported into cells by clathrin-dependent endocytosis. (A) Representative pictures of the transport of FITC-labeled rGSTP1 into Hepa1-6 cells. (B) Representative pictures of the intracellular localization of FITC-labeled rGSTP1 in Hepa1-6 cells after the treatment of different endocytosis inhibitors. Scale bar, 20 μm.

Table S1 Information of materials used in the present study

**Antibodies**		
**Antibodies**	**Vendor**	**Catalog number**
G9a	Abcam (for WB)	31874
G9a	Abcam (for ChIP)	185050
GLP	Bioss	16789
H3K9me2	Abcam	1220
H3K9me1	Abcam	9045
Histone H3	Cell Signaling	9715
HSP70	BD Biosciences	03719
HSP90	BD Biosciences	03717
VCAM-1	Santa Cruz	8304
ICAM-1	Santa Cruz	1511
MCP-1	Santa Cruz	1785
iNOS	BD Biosciences	610431
eNOS	BD Biosciences	610298
3-Nitrotyrosine	Millipore	MAB5404
p-Chk2	Cell Signaling	2197
p-Chk1	Cell Signaling	2348
p-p53	Cell Signaling	9284
p53	Santa Cruz Bio	99
p-H2A.X	Cell Signaling	9718
PARP-1	ENZO Life Sciences	ALX-804-211
Caspase3	Cell Signaling	9662
Caspase8	Beyotime	AC056

GSTP1	Santa Cruz	28494
Nrf2	Santa Cruz (for WB)	365949
Nrf2	Cell Signaling (for IHP)	12721
Keap1	Santa Cruz	33569
F4/80	Santa Cruz	52664
Ly-6G	BD Biosciences	551459
CD3	Gene Tech	GA045229
8-oxoG	Santa Cruz	130914

qPCR primers

Genes	Species	Sequences
Ehmt2	*Mouse*	Forward: TCGGGCAATCAGTCAGACAG
		Reverse: TGAGGAACCCACACCATTCAC
Ehmt1	*Mouse*	Forward: GAACAGGAGTCTCCCGACAC
		Reverse: GGGCTGTCAGTCTTCCCTC
Tnfa	*Mouse*	Forward: GGTGATCGGTCCCCAAAGGGATGA
		Reverse: TGGTTTGCTACGACGTGGGCT
Il1b	*Mouse*	Forward: GAAGAAGAGCCCATCCTCTG
		Reverse: TCATCTCGGAGCCTGTAGTG
Il6	*Mouse*	Forward: CACTTCACAAGTCGGAGGCT
		Reverse: CTGCAAGTGCATCATCGTTGT
Mcp-1	*Mouse*	Forward: GCTCTCTCTTCCTCCACCAC
		Reverse: GCGTTAACTGCATCTGGCT
Atf3	*Mouse*	Forward: CTCTCACCTCCTGGGTCACT
		Reverse: TCTGGATGGCGAATCTCAGC
Cox2	*Mouse*	Forward: CAACACCTGAGCGGTTACCA

		Reverse: TTCAGAGGCAATGCGGTTCT
Hmox1	*Mouse*	Forward: CCTCACAGATGGCGTCACTT
		Reverse: GCTGATCTGGGGTTTCCCTC
Gstp1	*Mouse*	Forward: GCGGCAAATATGTCACCCTCAT
		Reverse: GCAGGGTCTCAAAAGGCTTC
Gstp2	*Mouse*	Forward: ATATGGCACCATGATCTACAGAAA
		Reverse: TCTGGTCACCCACGATGAAA
Nrf2	*Mouse*	Forward: CCTCTGTCACCAGCTCAAGG
		Reverse: TTGATGACCAGGACTCACGG
Keap1	*Mouse*	Forward: GTGGCACCTACAGAGACACC
		Reverse: GCGCTTGGAGAAGGGCA
Nqo1	*Mouse*	Forward: CATTGCAGTGGTTTGGGGTG
		Reverse: TCTGGAAAGGACCGTTGTCG
Gcl	*Mouse*	Forward: AGCTTTGGGTCGCAAGTAGG
		Reverse: GGTCTCGCGCTCCCAC
Mrp1	*Mouse*	Forward: CAAAGCCGGTGGAAAATGGG
		Reverse: GTGGGAAGACGAGTTGCTGA
Sod2	*Mouse*	Forward: TAACGCGCAGATCATGCAGCTG
		Reverse: AGGCTGAAGAGCGACCTGAGTT
Cat	*Mouse*	Forward: TGAGAAGCCTAAGAACGCAATTC
		Reverse: CCCTTCGCAGCCATGTG
Trx1	*Mouse*	Forward: GAAATGCATGCCGACCTTCCAG
		Reverse: GCTGGTTACACTTTTCAGAGCATG
Gpx2	*Mouse*	Forward: TGTGACGTCAATGGGCAGAA
		Reverse: GTCATGAGGGAGAACGGGTC

β-actin	*Mouse*	Forward: GGCTACAGCTTCACCACCAC<br>Reverse: TAGCTCTTCTCCAGGGAGGA
**ChIP-qPCR primers**		
Name	promoter region (+1 for TSS)	Sequences
Gstp1-1591	-1591 to -1506	Forward: AAGGGGGCCCCATGTTGA<br>Reverse: ATCCAAAGCCAGGGAATTAGCTCTG
Gstp1-1437	-1437 to -1344	Forward: ACCTTGCTGGTGATACGTGAG<br>Reverse: CTCCTCGGTGTCTTACTTACTCTG
Gstp1-898	-898 to -751	Forward: TGGCCTGTTTAAAGGTGGAGAA<br>Reverse: TGTGAGAATAGGGACAAATCTTGAG
Gstp1-473	-473 to -375	Forward: TCCAACCCTGTTAGTCTCGTTT<br>Reverse: CCTTCGCCAGGAGTAATCAG
Gstp2-1904	-1904 to -1832	Forward: GCCCAGAACCTCAGTGATGAA<br>Reverse: TGCACTGTAGCGTCACATTCT
Gstp2-1094	-1094 to -1014	Forward: TGCGGGAGGAAAGAGTGAGA<br>Reverse: GTGTCTCCCTGGGTAATGGG
Gstp2-158	-158 to -40	Forward: ACATGCTAGTGTCTCGGGC<br>Reverse: TATAAGGGGCATTGCGCTC
Tnfa-1753	-1753 to -1669	Forward: TTCTTTGGAGCCTTTGCACT<br>Reverse: ACCCTTGAAACAACGGTCAG
Tnfa-1486	-1486 to -1329	Forward: GGACATGAAGGAGGGACAGA<br>Reverse: CACCTTTGTGTCTGGGACCT
Tnfa-768	-768 to -523	Forward: CGCAGTCAAGATATGGCAGA<br>Reverse: CTTGGAGGAAGTGGCTGAAG

Il1b-1995	-1995 to -1823	Forward: CTCCAAATCCTCCCAGACAA
		Reverse: AAGGGTAACTAGGGGCCTGA
Il1b-1483	-1483 to -1431	Forward: ATAGCTGGTCAAAGGCAGGA
		Reverse: GCATCTCGATTTCAGGAAGG
Il1b-608	-608 to -512	Forward: AAGCACCCTGGCATTATCAG
		Reverse: AGGGCACACACAGTATGCAG
Il6-1634	-1634 to -1502	Forward: CACACGGTGAAAGAATGGTG
		Reverse: AAAGCCGGTTGATTCTTGTG
Il6-1142	-1142 to -1038	Forward: GGTGGACAGAAAACCAGGAA
		Reverse: TAACCCCTCCAATGCTCAAG
Il6-632	-632 to -527	Forward: TGCACAAAATTTGGAGGTGA
		Reverse: ACCCAACCTGGACAACAGAC

TSS, transcription starting site.
